# Evolutionary gradient of predicted nuclear localization signals (NLS)-bearing proteins in genomes of family Planctomycetaceae

**DOI:** 10.1186/s12866-017-0981-y

**Published:** 2017-04-04

**Authors:** Min Guo, Ruifu Yang, Chen Huang, Qiwen Liao, Guangyi Fan, Chenghang Sun, Simon Ming-Yuen Lee

**Affiliations:** 1 State Key Laboratory of Quality Research of Chinese Medicine and Institute of Chinese Medical Sciences, University of Macau, Macao, China; 2 State Key Laboratory of Pathogen and Biosecurity, Beijing Institute of Microbiology and Epidemiology, Beijing, China; 3 Department of Microbial Chemistry, Institute of Medicinal Biotechnology, Chinese Academy of Medical Sciences & Peking Union Medical College, Beijing, China

**Keywords:** Planctomycetaceae, Comparative genomics, Nuclear localization signal, Signal peptide transformation

## Abstract

**Background:**

The nuclear envelope is considered a key classification marker that distinguishes prokaryotes from eukaryotes. However, this marker does not apply to the family Planctomycetaceae, which has intracellular spaces divided by lipidic intracytoplasmic membranes (ICMs). Nuclear localization signal (NLS), a short stretch of amino acid sequence, destines to transport proteins from cytoplasm into nucleus, and is also associated with the development of nuclear envelope. We attempted to investigate the NLS motifs in Planctomycetaceae genomes to demonstrate the potential molecular transition in the development of intracellular membrane system.

**Results:**

In this study, we identified NLS-like motifs that have the same amino acid compositions as experimentally identified NLSs in genomes of 11 representative species of family Planctomycetaceae. A total of 15 NLS types and 170 NLS-bearing proteins were detected in the 11 strains. To determine the molecular transformation, we compared NLS-bearing protein abundances in the 11 representative Planctomycetaceae genomes with them in genomes of 16 taxonomically varied microorganisms: nine bacteria, two archaea and five fungi. In the 27 strains, 29 NLS types and 1101 NLS-bearing proteins were identified, principal component analysis showed a significant transitional gradient from bacteria to Planctomycetaceae to fungi on their NLS-bearing protein abundance profiles. Then, we clustered the 993 non-redundant NLS-bearing proteins into 181 families and annotated their involved metabolic pathways. Afterwards, we aligned the ten types of NLS motifs from the 13 families containing NLS-bearing proteins among bacteria, Planctomycetaceae or fungi, considering their diversity, length and origin. A transition towards increased complexity from non-planctomycete bacteria to Planctomycetaceae to archaea and fungi was detected based on the complexity of the 10 types of NLS-like motifs in the 13 NLS-bearing proteins families.

**Conclusion:**

The results of this study reveal that Planctomycetaceae separates slightly from the members of non-planctomycete bacteria but still has substantial differences from fungi, based on the NLS-like motifs and NLS-bearing protein analysis.

**Electronic supplementary material:**

The online version of this article (doi:10.1186/s12866-017-0981-y) contains supplementary material, which is available to authorized users.

## Background

Species in the Planctomycetaceae family are ecologically widespread, including even human gut and blood but ubiquitous in water and soil [1–8]. Although Planctomycetaceae are taxonomically affiliated with bacteria, they have been in past studies reported to possess a number of characteristics that are closer to eukaryotes, especially the absence of peptidoglycan in their cell envelope, synthesis of membrane sterols and the presence of membrane-coat proteins [1, 9–11]. Another intriguing characteristic of Planctomycetaceae is their cellular compartmentalization due to development of internal lipid intracytoplasmic membranes (ICMs) [1, 12], which is uncommon in prokaryotes. In the Planctomycetaceae family, only *Gemmata obscuriglobus* has double-layer ICMs [12]; the other species of the family contain single-layer ICMs. Nevertheless, recently, species in this family have been experimentally confirmed to contain peptidoglycan in their cell wall [13, 14]. Moreover, nearly all of the “unique” characteristics beyond non-planctomycete bacteria in Planctomycetaceae have been argued not relevant to homology with eukaryotic characteristics, with many of them proposed to result from convergent evolution or lateral gene transfer [15]. Arguing on the other hand in favor of potential homology is the finding that ICMs divide cells of all examined planctomycete species into two compartments, the paryphoplasm and pirellulosome [16, 17], and consequently may make transcription and translation independent, allowing for the development of eukaryotic cellular complexity [18]. The exact nature and topology of planctomycete cell compartments has been subject to controversy, and the question of a closed nucleoid-associated membrane envelope is especially subject to debate [19, 20] - compartments completely closed by membranes may however imply some form of transport system similar to that used by eukaryotes for nucleocytoplasmic transport. A study of the cellular compartmentalization of *G. obscuriglobus* using an immunogold approach found a substantial difference from *Escherichia coli* in the distribution of FtsK protein, which may give insights into the origin of the eukaryotic endomembrane system [20, 21]. Thus, exploration of unusual molecular features that may contribute to or be a consequence of the complicated internal features of family Planctomycetaceae is urgent.

A eukaryotic nucleus has complicated structural and functional foundation, particularly the nuclear pore complex (NPC) [22], a component of the nuclear envelope, which is involved in communication of macromolecules over 60 KDa between the nucleoplasm and cytoplasm. Two types of short amino acids stretches are the signals that direct the transport of macromolecules through the NPC: nuclear location signals (NLSs) [23] and nuclear export signals (NESs) [24, 25]. With other potential cellular functions [26], NLSs direct molecular transport from the cytoplasm to nucleoplasm, and NESs direct transport in the opposite direction. NES motifs are leucine (L) rich and NLSs are arginine (R) and lysine (K) rich. NLS motifs are monopartite or bipartite [27] and their location and number in proteins can vary. NLSs and NESs have been widely identified in many organisms as conferring the ability on a protein to shuttle through the nuclear membrane [28, 29]. Only a few cytoplasmic proteins without a typical NLS core peptide enter the nucleus and they do this only via a strong interaction with protein factors with a core NLS motif [30]. The intracellular environment is crucial to the function of NLS and NES motifs [31]. NLS or NES motifs generally need to be exposed at the protein surface to bind to importins or exportins. Thus, the cell needs mechanisms to unmask hidden or cryptic NLS or NES motifs in proteins; these mechanisms include phosphorylation or dephosphorylation, dissociation of an inhibitory subunit that masks the NLS, processing of a larger precursor, and binding of hormones at a certain stage of development [31]. An NLS database (http://rostlab.org/services/nlsdb/browse.php) has 114 experimentally identified NLS motifs to date [23, 32].

Earlier reported experimental studies of bacterial NLS sequences demonstrated in *Thermoplasma* [33, 34], *Streptomyces*, and *Agrobacterium* [35] the functionality of prokaryotic NLS in transporting proteins into a eukaryotic nucleus. However, no genomic or experimental investigation of NLS motif or NLS-bearing proteins has so far been reported in Planctomycetaceae [36]. Considering the complicated cellular membrane structures of Planctomycetaceae species and the critical functional role played by NPCs and the correlated NLS-sequences in proteins destined for transporting into the nucleus, herein we aim to determine the status of NLSs and NLS-bearing proteins in the Planctomycetaceae family and other microorganisms by a comparative genomic approach. The analysis of signals in Planctomycetaceae related in eukaryotes to the existence of a nuclear envelope (and functions of which might be expected to be absent in bacteria) may help in understanding the underlying stages in molecular evolution correlating with the origin of cell structure complexity.

## Methods

### Data normalization

In order to evaluate the significance of transformation of NLS-like motifs among bacteria, Planctomycetaceae and fungi groups, index Q value was developed, in which the sizes of protein pool and genome, and gene amount were considered for normalization, defined as:

$$ {\mathrm{Q}}_{\mathrm{i}}=\raisebox{1ex}{${\mathrm{M}}_{\mathrm{i}}$}\!\left/ \!\raisebox{-1ex}{$1\mathrm{g}\left({\mathrm{N}}_{\mathrm{i}}*{\mathrm{G}}_{\mathrm{i}}\right)$}\right. $$

Where M_i_ is the NLS-like motif abundance in ith species, and N_i_ and G_i_ are the gene amount and genome size of the ith species respectively. The larger Q value, the more NLS-like motifs harbor in the ith species.

### Principal component analysis

Covariance analysis used software CanoDraw for Windows 4.0 (http://www.canodraw.com/) with diagrams processed in Adobe Illustrator CS6 [37].

### Ortholog retrieval

Orthologs were determined using software OrthoMCL [38]. At first, this program conducts an all-against-all BLASTp search in BLAST 2.2.25. OrthoMCL then converts the reciprocal BLAST p-values to a normalized similarity matrix that is analyzed using a Markov Cluster algorithm (MCL). This yields many clusters, each containing a set of orthologs and/or recent paralogs. The BLAST e-value cut-off was ≤1e^−5^; other parameters were defaults.

### Evaluation of the complexity of NLSs-like motifs

We generated a score matrix considering diversity, length and origin of NLS-like motifs (Table 1). We measured the complexity of the NLS motif from two aspects: the length and diversity of the motif (in structure), and the evolutionary origin of the motif (in evolution). We calculated scores with simple conversions or formula based on the appearance/abundance of the motif in the 27 genomes, and the methods (conversion and formula) were also described there (Table 1).

**Table 1:** Score matrix of the 10 NLS-motifs in the 13 NLS-bearing protein families, considering length, diversity and origin of NLS-like motifs

NLS motif	length	diversity	origin
PRRRK	5	3	5.66
RKRKK	5	3	5.98
RPRRK	5	3	5.38
KRPRP	5	3	5.36
RKRRR	5	2	7.96
P.KKKRK	7	4	5.3
TKRS…M	8	11	5.14
KR.{10}KKKL	16	23	5.12
K[RK]{3,5}.{11,18}[RK]K.{2,3}K	25	38	8.54
[KR]{4}.{20,24}K{1,4}.K	31	58	11.4

Note: ‘length’ means total amino acid number in NLS motif; ‘diversity’ refer to total amino acid types in a NLS motif, symbols as ‘.’ or ‘[]’ in NLS motif will be given an extra reward score (+1, + 0.5 respectively); ‘origin’ was used here to judge the potential evolutionary dominance of NLS motif through classification of the species accommodating the NLS motif. Taking Planctomycetaceae as control (score = 0), if one NLS motif is only found in non-planctomycete bacteria but not found in eukaryotic or Planctomycetaceae species, this NLS motif will be regarded as having low evolutionary dominance, and its scored will be punished (− 5) regardless of NLS motif abundance in these species. In contrast, if one NLS motif was detected in a eukaryotic species, it will obtain a reward score (+5), meanwhile, if the average abandance of the NLS motif in a eukaryotic species is high, that means, this NLS motif will have much higher evolutionary dominance, and it will be given an extra reward score (reward score = average abundance of the motif in eukaryotes/10)

### NLS-bearing proteins abundance in the 27 strains

We obtained all 114 experimentally identified NLS motifs from NLS database (http://rostlab.org/services/nlsdb/browse.php). After searching the 114 NLS motifs in 27 genomes, we obtained 1101 NLS-proteins (Additional file 1: Table S1, A), and generated a heat-map with R software (version 2.13.0).

### Function annotation and metabolic pathway analysis

Functions of NLS-bearing protein families were assigned using the best match of the alignments using BLASTp (E-value ≤ 10–5) searching against the SwissProt (Release 15.10) [39] and KEGG databases (Release 48.2) [40]. If the best hit of the proteins with any of these processes was “function unknown,” or “putative,” second-best hits were used to assign function until no additional hits met the alignment criteria. Analysis of metabolic pathways was performed by ipath 2.0 (http://pathways.embl.de/) using the assigned KO numbers in KEGG Orthology system.

## Results

### NLS-like motifs in the family Planctomycetaceae

**Fig. 1:**
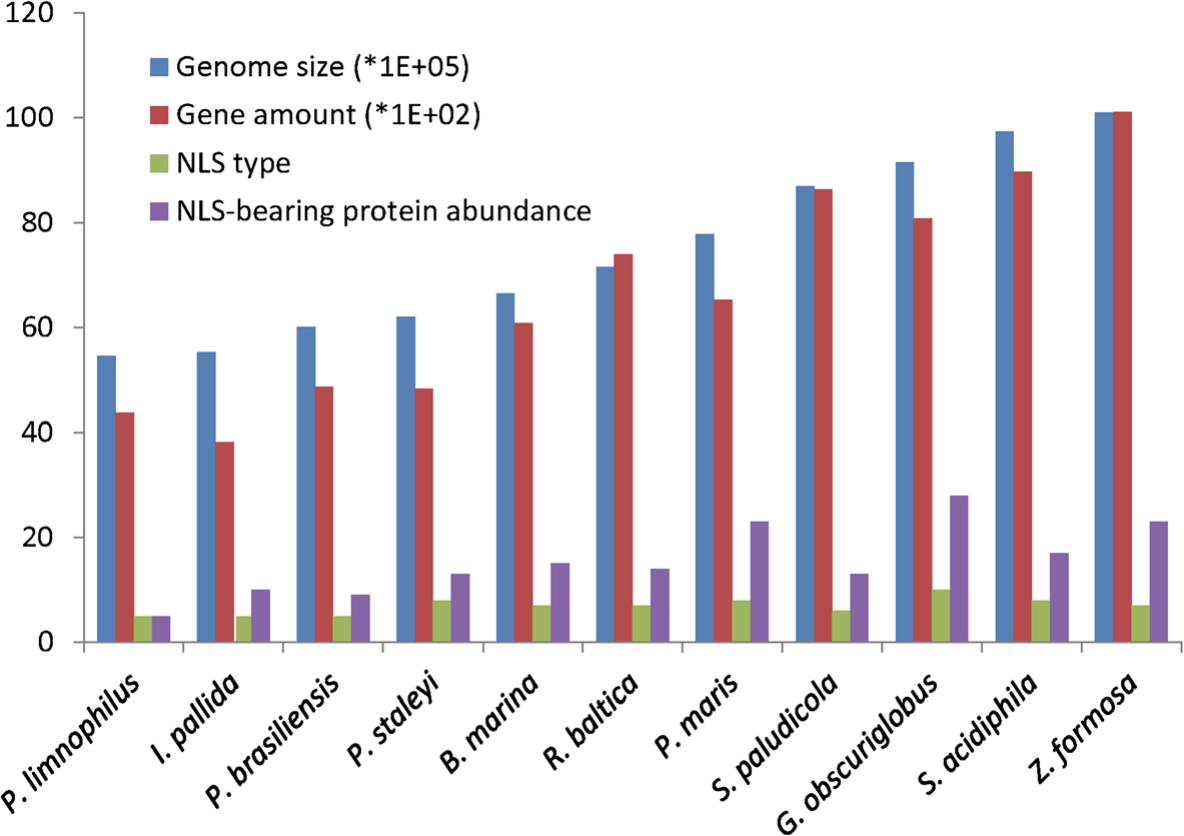
Genome size, gene amount, NLS-like motif types and NLS-bearing protein abundance in genomes of the 11 Planctomycetaceae strains

To date, 114 experimentally identified NLS motifs are in the NLS database (http://rostlab.org/services/nlsdb/browse.php). After searching protein pools of the 11 Planctomycetaceae species using amino acid sequences of the 114 NLS motifs, a total of 15 NLS types and 170 NLS-bearing proteins were detected in the family Planctomycetaceae. We arranged the order of the 11 species in Planctomycetaceae on the basis of genome size (Fig. 1). Multiple regression analysis indicated that NLS type or NLS-bearing proteins abundance express insignificant correlations with genome size or gene amount (*P* > 0.05). However, the double-layer ICMs strain *G. obscuriglobus* had the most abundant NLS-bearing proteins (28) and the most NLS types (10) in the family Planctomycetaceae. Both the NLSs KR.{10}KKKL (the dot means any amino acid; the number in brace means copy number) and KAKRQR were seen and the highest frequency of RKRRR was observed in *G. obscuriglobus* compared to other strains in the family.

### NLS-bearing protein abundance of 27 strains

To better illustrate the relative distribution and abundance of NLS and NLS-bearing proteins in Planctomycetaceae relative to other groups of bacteria and eukaryotes, as judged by comparative genomics, to the analysis of 11 strains of Planctomycetaceae we added 16 extra representative microbes from different microbial taxonomical communities and retrieved their genomes from NCBI database (ftp://ftp.ncbi.nlm.nih.gov/genomes/). Phylogenetic relatives of Planctomycetaceae [41], especially two members of the Planctomycetaceae-Verrucomicrobia-Chlamydiae (PVC) superfamily [1], were included in the analysis for comprehensive phylogenetic representation. Through searching the 27 predicted protein pools (the 11 Planctomycetaceae strains and the other 16 microorganisms) using the 114 identified NLS motifs, we discovered 29 NLS types and 1101 NLS-bearing proteins (Additional file 1: Table S1, A). For the 29 NLS motifs, 15 NLS types were detected in the family Planctomycetaceae, and the rest of the NLS types were discovered in eukaryotes. ‘QRKRQK’ was only found in non-planctomycete bacteria and eukaryotes; ‘RRKGKEK’ and ‘KRKRRP’ were only found in Planctomycetaceae.

**Fig. 2:**
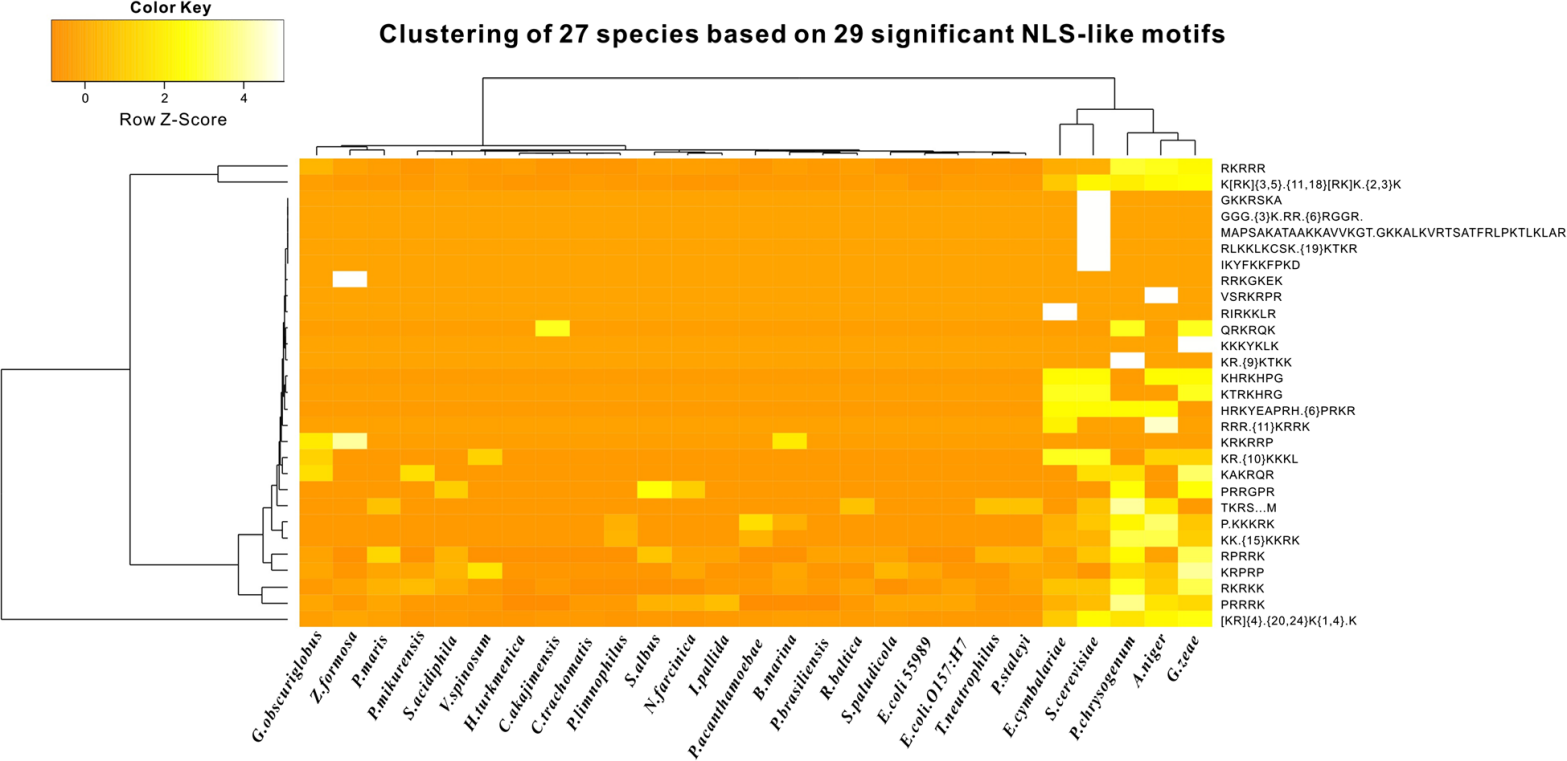
Abundance of NLS-bearing proteins with the 29 types of NLS-like motifs in the 27 predicted protein pools. The 27 species contains 9 bacteria (*N. farcinica*, *S. albus*, *E. coli* O157, *E. coli*, *C. akajimensis*, *P. acanthamoebae*, *P. mikurensis*, *C. trachomatis*, *V. spinosum*), 11 Planctomycetaceae strains (*Z. Formosa*, *S. acidiphila*, *S. paludicola*, *R. baltica*, *P. maris*, *P. limnophilus*, *P. brasiliensis*, *P. staleyi*, *I. pallida*, *B. marina*, *G. obscuriglobus*), 2 archaea (*T. neutrophilus*, *H. turkmenica*) and 5 fungi (*E. cymbalariae*, *S. cerevisiae*, *A. niger*, *P. chrysogenum*, *G. zeae*). The phylogenetic tree on the top of the figure shows correlations of the 27 strains. The tree on the left shows phylogenetic correlations of the 29 types of NLS-like motifs. Color bar shows the abundance of NLS-bearing proteins on the right

Correlations between the 27 strains were shown by the occurrence frequencies of NLS-bearing proteins with the 29 types of NLS-like motifs in their predicted protein pools (Fig. 2). Phylogenetically, the 27 strains were divided approximately into two branches. The first branch includes bacteria, Planctomycetaceae, and archaea; the second contains only fungi. In Fig. 2, eukaryotic organisms possessed more NLS-bearing proteins and frequently had longer and more diverse NLS-like motifs than bacteria, and prokaryotes tended to have simple and short NLS-like motifs. However, many short, simple NLS-like motifs were still widely found in fungi (Additional file 1: Table S1, A). We hypothesized that some short and simple NLS-like motifs were inherited from an evolutionary ancestor, before activation of their function. Afterward, these motifs were first activated and extensively used in NLS-bearing proteins from the perspective of evolutionary economics of energy consumption. Some longer and more complicated NLS-like motifs then appeared in eukaryotic species to meet higher or special demands of intracellular molecular communication. Our results were consistent with this hypothesis, as shown in Fig. 2, the PRRRK, RKRKK, KRPRP and RPRRK NLS-like motifs, which appeared in bacteria (including non-planctomycete bacteria and planctomycete bacteria), were dramatically increased in fungi. By contrast, the GKKRSKA, IKYFKKFPKD, and K[RK]{3,5}x{11,18}[RK]Kx{2,3}K (where x is any amino acid; the characters in bracket means alternative) motifs only occurred in fungi. Curiously, the bipartite NLS-like motif [KR]{4}x{20,24}K{1,4}xK was found in all the 27 strains. We attributed the emergence of long bipartite NLS-like motif in non-planctomycete bacteria to the high plasticity of this NLS-like motif regardless of its length.

**Fig. 3:**
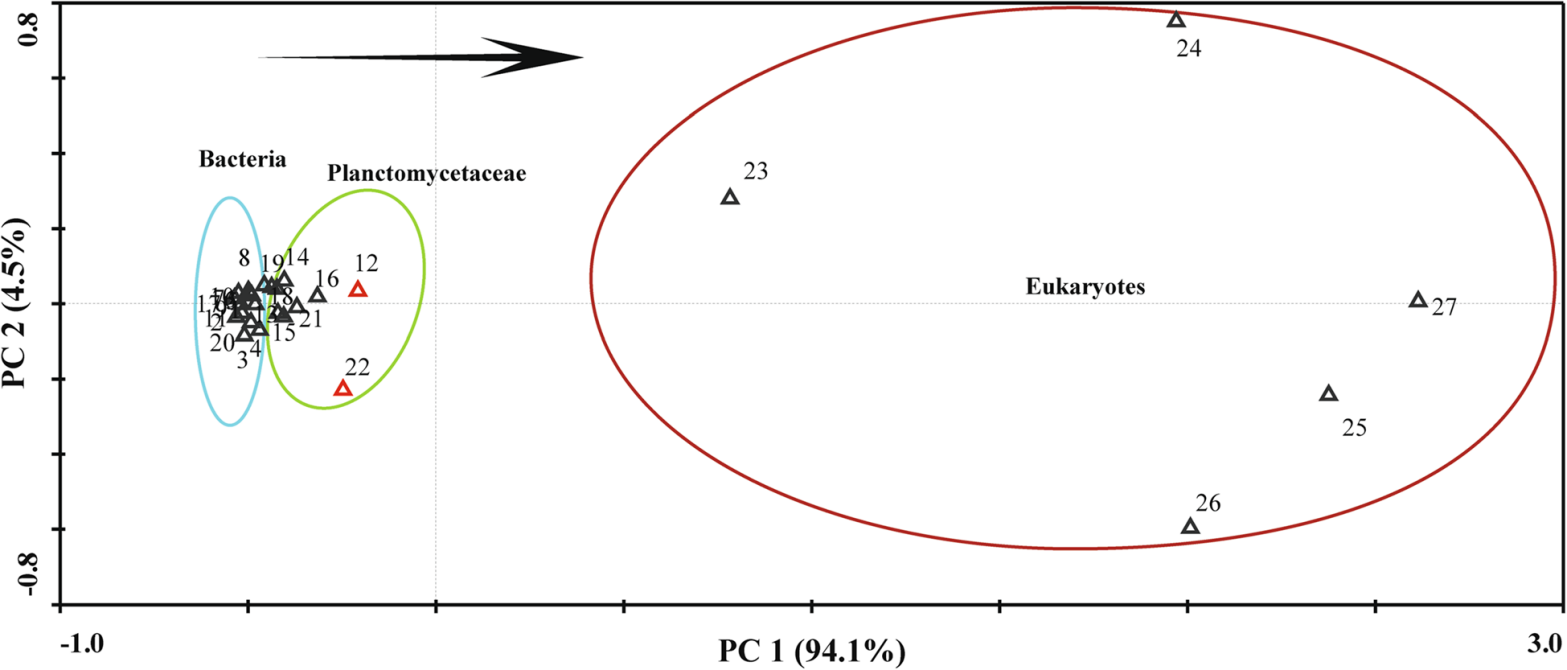
PCA of the 27 strains. Distance between up-triangles approximates dissimilarity of abundance profiles of the NLS-bearing proteins in the 27 strains, measured by euclidean distance. Red, up-triangles show *G. obscuriglobus* (12) and *Z. formosa* (22). Numbers in the figure indicate: 1: *T. neutrophilus*; 2: *H. turkmenica*; 3: *N. farcinica*; 4: *S. albus*; 5: *E. coli* O157; 6: *E. coli*; 7: *C. akajimensis*; 8: *P. acanthamoebae*; 9: *P. mikurensis*; 10: *C. trachomatis*; 11: *V. spinosum*; 12: *Z. formosa*; 13: *S. acidiphila*; 14: *S. paludicola*; 15: *R. baltica*; 16: *P. maris*; 17: *P. limnophilus*: 18: *P. brasiliensis*; 19: *P. staleyi*; 20: *I. pallida*; 21: *B. marina*; 22: *G. obscuriglobus*; 23: *E. cymbalariae*; 24: *S. cerevisiae*; 25: *A. niger*; 26: *P. chrysogenum*; 27: *G. zeae*

After normalizing the data of NLS-bearing proteins abundance in the 27 genomes considering genome size and protein quantity (Additional file 1: Table S1, B), we detected a significant correlation between the 27 strains. Principal component analysis showed a significant transitional gradient (revealed by euclidean distance: planctomycete groups displayed a shorter euclidean distance to eukaryotic groups than non-planctomycete bacteria, Fig. 3) from bacteria to Planctomycetaceae to fungi in NLS-bearing protein abundances. Planctomycetaceae species separated slightly from bacteria, but were substantially distinguished from fungi. Remarkably, two Planctomycetaceae species, *Z. formosa* and *G. obscuriglobus* stand closest to eukaryotes (Fig. 3, in red up-triangles).

### Clustering and annotation of NLS-bearing proteins

**Fig. 4:**
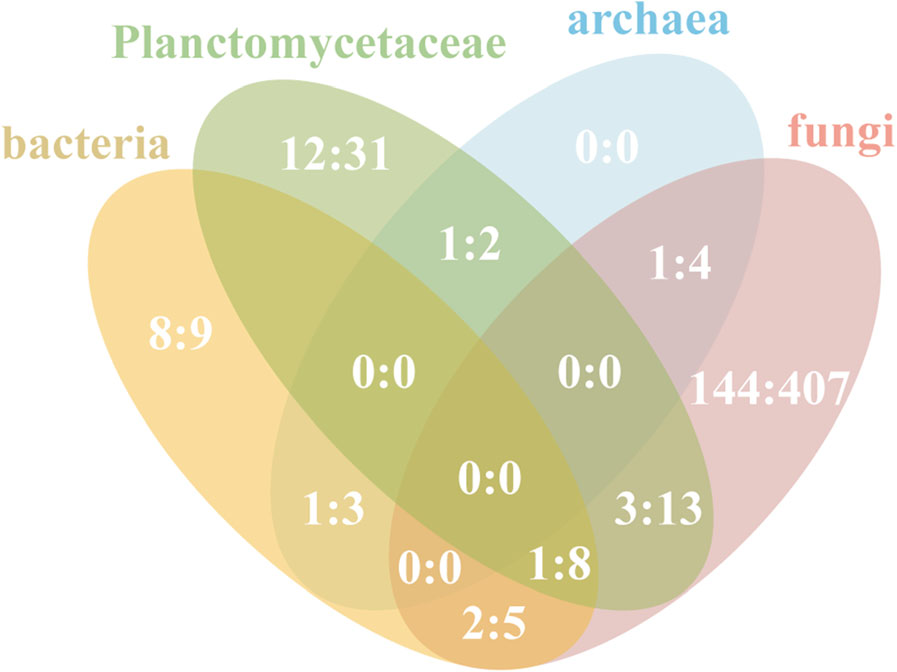
Clustering of the 993 NLS-bearing proteins in the 27 strains. The figures in venn diagram indicate family quantity. Each area of the venn diagram contains two figures divided with a semicolon. The *left* number indicates the NLS-bearing protein family quantity and the *right* indicates NLS-bearing protein quantity in these families

We used the 993 non-redundant NLS-bearing proteins instead of all the 1101 NLS-like proteins for clustering and functional annotation. Shared Protein families of all 993 nonredundant NLS-like proteins are showed by venn diagram (Fig. 4), excluding orphan proteins. Fungi possessed the most NLS-bearing protein families and NLS-bearing proteins, but shared a very small number of them with Planctomycetaceae (four families) or bacteria (three families). Planctomycetaceae and bacteria shared more NLS-bearing protein families (nine families) [42]. The five fungal strains have as many as 144 unique NLS-bearing protein families. By contrast, bacteria have only eight unique NLS-bearing protein families, and Planctomycetaceae have 12 unique NLS-bearing protein families.

There were 727 NLS-bearing proteins were annotated in SWISS-PROT database (Additional file 1: Table S2), but only 537 were annotated in Kyoto Encyclopedia of Genes and Genomes (KEGG) database (Additional file 1: Table S3). We aligned the eight homologous NLS-bearing proteins of the only one family shared among bacteria, Planctomycetaceae and fungi (Additional file 2: Dataset S1).

**Fig. 5:**
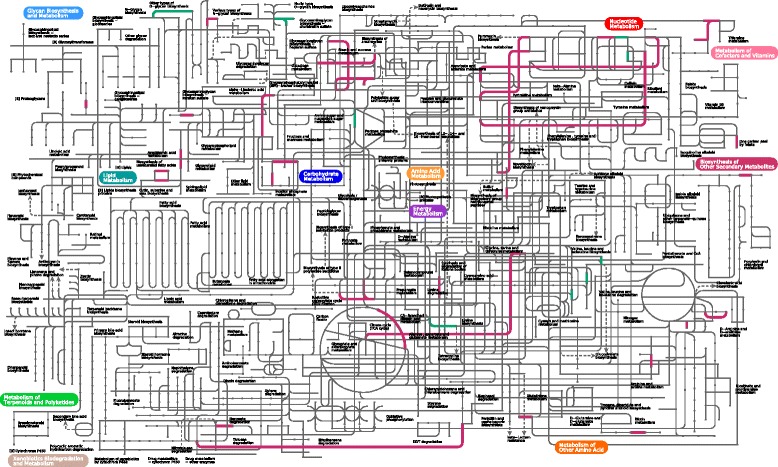
Metabolic pathways of NLS-bearing proteins of Planctomycetaceae and fungi. Pathways colored *pinkish red* show NLS-bearing proteins of fungi; Pathways colored *green* show NLS-bearing proteins of Planctomycetaceae

To better demonstrate the functional evolution of real NLS motifs, we investigated core/pan metabolic pathways using the annotated NLS-bearing proteins of Planctomycetaceae (49 NLS-bearing proteins) and fungi (457 NLS-bearing proteins). A total of 66 metabolic pathways were referred, in which fungi occupied 57 metabolic pathways. In Fig. 5, NLS-bearing proteins of Planctomycetaceae preliminarily stepped in a range of basic material metabolism, such as sulfates [43], O/N-glycan biosynthesis and metabolism, hydrophobic amino acid (valine, leucine and isoleucine) biosynthesis and purine metabolism. NLS-bearing proteins of fungi notably reinforced the pathways Planctomycetaceae’s NLS-bearing proteins referred and extended the scopes to complex compound metabolism, particularly degradation of benzoate and its derivatives. Likewise, fungi’s NLS-bearing proteins shared with more regulatory pathways than Planctomycetaceae, NLS-bearing proteins of which mainly serve in ribosome and RNA degradation (Additional file 2: Figure S1). Interestingly, we found traces of Planctomycetaceae’s NLS-bearing proteins on protein export.

### Transformation of NLS motifs in NLS-bearing protein families

**Fig. 6:**
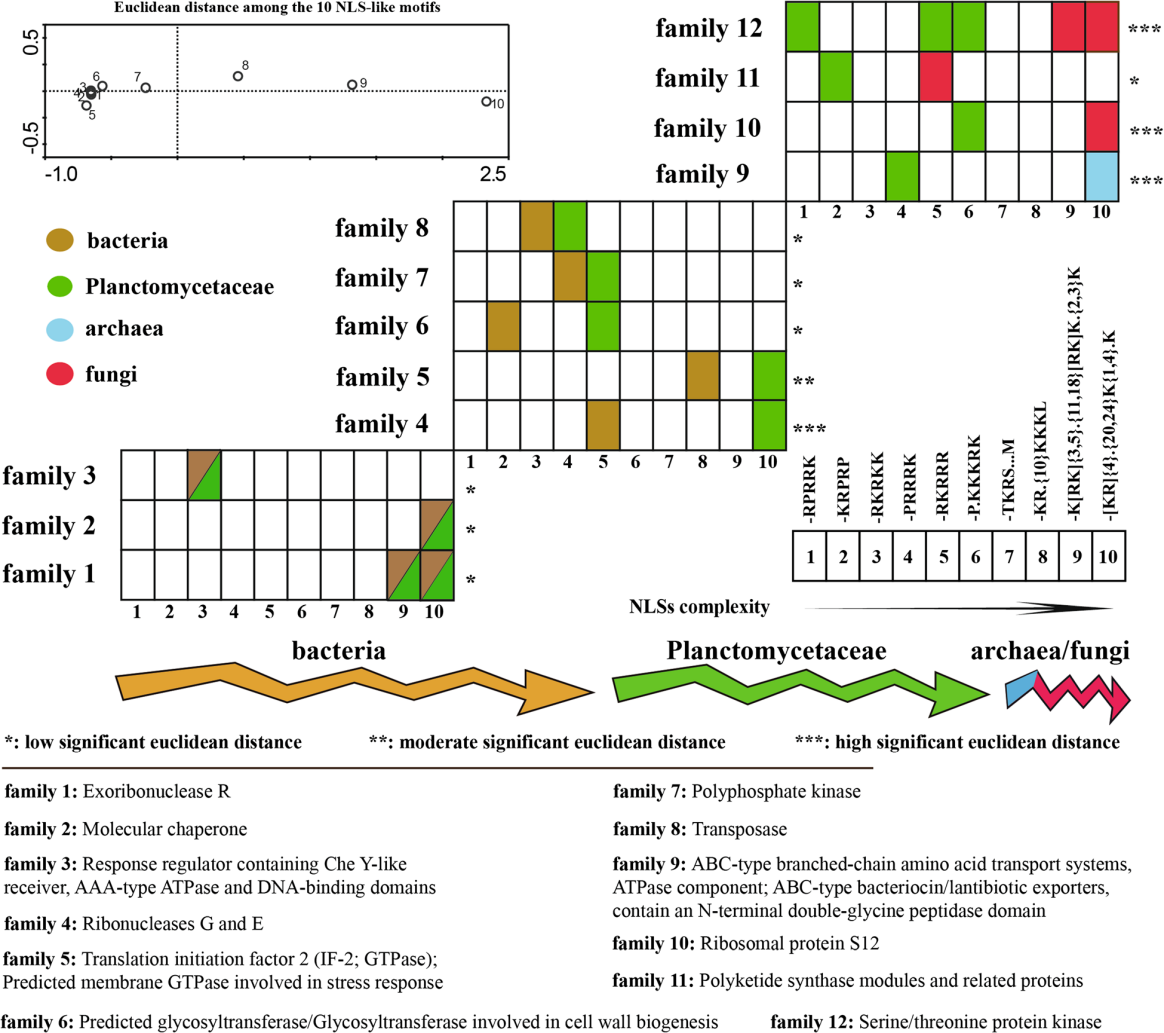
Analysis of the 13 common NLS-bearing protein families among bacteria, Planctomycetaceae, archaea or fungi. There are 10 types of NLS-like motif in the 13 families in all. As shown in the figure, each family bar contains 10 small patches indicating one of the 10 NLS-like motif types. Euclidean distances of the 10 NLS-like motifs show at up and left. The 10 types of NLS-like motif arranged in order of simple to complex in family bars. Colors of responded patches in the family bars match microbial community colors below the euclidean distance map of the 10 NLS-like motifs, up and left, meaning the types of NLS-like motif in the patches derived from NLS-bearing protein of the community. Asterisks beside the family bar indicate the significance of NLS-like motif type change in family according to euclidean distance. Functions of the 13 NLS-bearing protein families show at bottom

To explore the potential transformations of NLS-like motifs in NLS-bearing protein families, we picked out 13 common NLS-bearing proteins families among bacteria, Planctomycetaceae, archaea or fungi for further analysis (Additional file 2: Dataset S1). The 13 NLS-bearing protein families contained 42 NLS-bearing proteins and ten types of NLS-like motif. We arranged the ten types of NLS-like motifs from simple to complex, considering their diversity, length and origin (Table 1). Consequently, the 13 NLS-bearing protein families were divided into three groups (Fig. 6). The first group contained three NLS-bearing protein families that are common to bacteria and Planctomycetaceae and harbored proteins with the same types of NLS-like motif. The second group contained five NLS-bearing protein families that are common to bacteria and Planctomycetaceae and harbored proteins with different types of NLS-like motif. The third group contained five NLS-bearing protein families that are common to Planctomycetaceae and archaea or fungi and harbored proteins with different types of NLS-like motif. Interestingly, Planctomycetaceae showed small and large significant changes compared with bacteria and fungi respectively, based on analyzing complexity of the ten NLS-like motifs in the 13 NLS-bearing protein families among bacteria, Planctomycetaceae, archaea or fungi. This result also point towards to presence of another “a-small-step-forward” genomic change in Planctomycetaceae species along the transformational gradient (Fig. 6). In Fig. 6, the first group accommodating proteins with common NLS-like motif(s) are three families between Planctomycetaceae strains and *Parachlamydia acanthamoebae* (family 2), *Verrucomicrobium pinoum* (family 3) or *Chlamydia trachomatis* (family 1). All of these species belongs to the PVC superfamily [1] [44]. Likewise, *V. pinoum* created low significant changes on NLS-like motif complexity with Planctomycetaceae members (family 5 and 6). Although *Phyciphaera mikureni* is one of Planctomycetaceae relatives, it revealed significant changes on NLS-like motif complexity with *Z. formoa* (family 4), which was also supported by their significant euclidean distance in Fig. 3.

### NESs of the 27 strains

NESs are the functional counterparts to NLSs. NESs are leucine-rich stretches of 8 to 15 amino acids with regularly spaced hydrophobic residues that bind to the export karyopherin *CRM1*. La Cour et al. [25] published a NESbase (version 1.0) database with 75 entries with 80 experimentally determined NESs (http://www.cbs.dtu.dk/databases/NESbase-1.0/db.html). Xu et al. compiled an NES database that contained more than 230 experimentally validated leucine-rich NES-bearing *CRM1* cargoes [24, 45]. To investigate the proteins containing NES-like sequences in the 27 predicted protein pools, we collected 279 identified NES motifs that were sufficient to independently export a fused protein out of the nuclear envelope from the NES database constructed by Xu et al. [24]. The search identified only 14 NES-like proteins (Additional file 2: Dataset S2). These NES-bearing proteins were from fungi and were annotated as actin. Furthermore, few proteins in the 27 predicted protein pools perfectly matched the classical NES consensus sequence L-x(2,3)-[LIVFM]-x(2,3)-L-x-[LI] (where x represents any amino acid) [46].

## Discussion

Though intracellular compartments, for instance magnetosomes [47], acidocalcisomes [48], chromatophores [49], thylakoids [50] and endospores [51], were reported in specific non-planctomycete bacterial groups, the layout of intracellular compartmentalization of Planctomycetaceae species seem to be more close to eukaryotes in morphology, especially to *G. obscuriglobus* [1, 20] and *Z. formosa* [52]. *Z. formosa* has the largest genome length and coding sequences quantity, and similar to *G. obscuriglobus*, it shows more complicated cellular compartmentalization structures than other species of Planctomycetaceae. Besides, in phylogenetic trees built with conserved positions of ribosomal RNA [53] or feature frequency profiles of whole proteomes [54], Planctomycetales consistently displayed an ancient and independent origin distinct from non-planctomycete bacterial groups, which is topologically in accordance with occurrence of “a-small-step-forward” genomic/complexity change of NLS-like motifs of Planctomycetaceae species when compared with non-planctomycete bacteria.

A number of factors constrained this study. First, more than half of the 11 Planctomycetaceae genomes including *G. obscuriglobus* and *Z. formosa* remain incomplete; second, lots of KEGG Orthology (KO) numbers of NLS-proteins of Planctomycetaceae were excluded from the reconstructed metabolic pathways; third, few experimentally identified NLS/NES motifs deposited in existing databases narrowed genomic searching results of NLS-like motifs. The NLS-like motifs in bacteria may not have the same function as the corresponding eukaryotic NLS motifs. Eubacteria do not have functional NLS-bearing proteins because they do not have a nuclear envelope. The predicted NLS-like motifs in these domains are merely sequence similarities and intended to illustrate the transformational rules of the motif among bacteria, Planctomycetaceae, and fungi. Further studies are required to confirm if these NLS-like components in bacteria are direct functional precursors of the NLS-like motifs in Planctomycetaceae and fungi. In addition, although transcriptomic and proteomic studies of Planctomycetaceae species *Rhodopirellula baltica* (the first Planctomycetaceae species with its genome completely sequenced) have been reported [55–58], however, in perspective of organic evolution, there is still an urgent need transcriptomic and proteomic studies centering on *G. obscuriglobus* or *Z. formosa* in future.

## Conclusion

The genomic exploration of NLS-like motifs in species of family Planctomycetaceae provided us with insights into possible genomic changes contributing to the evolution of NLS and nuclear membranes. In the study, we focused on NLS-bearing proteins in 11 strains of the family Planctomycetaceae using comparative genomic approaches. We detected “a-small-step-forward” transitional gradients from non-planctomycete bacteria to Planctomycetaceae to fungi in abundance of NLS-bearing proteins or in complexity of NLS-like motifs evolved in the 13 clustered NLS-bearing protein families (presumable orthologous NLS-bearing proteins) in the 27 strains. The findings expanded our knowledge about the genomic features of family Planctomycetaceae and will facilitate understanding about the impact of NLS motifs in cellular development. The results suggest that a next step might be experimental test of function of NLS- sequences of planctomycetes within a eukaryote cell context (similar to past experiments with *Thermoplasma* and *Streptomyces*) and future experiments aimed at localizing NLS-bearing proteins in relation to cell compartments of *G. obscuriglobus* in particular may be informative.

## Additional files

Additional file 1: **Table S1 (A).** NLS-bearing protein abundance in the 27 strains; **Table S1 (B).** Normalized NLS-bearing protein abundance of the 27 strains. **Table S2.** Annotation of the 993 NLS-bearing proteins with SwissProt database. **Table S3.** Annotation of the 993 NLS-bearing proteins with SwissProt database. (XLSX 107 kb)

Additional file 2: **Figure S1.** Regulatory pathways of NLS-bearing proteins of Planctomycetaceae and fungi. Pathways colored pinkish red show NLS-bearing proteins of fungi; pathways colored green show NLS-bearing proteins of Planctomycetaceae; pathways colored light blue show the common Regulatory pathways between Planctomycetaceae and fungi. **Dataset S1.** The 13 clustered NLS-bearing protein families among non-planctomycete bacteria, Planctomycetaceae or fungi. **Dataset S2.** NES-bearing proteins in the predicted protein pools of the 27 strains. (PDF 408 kb)

## Abbreviations

A: Ala
C: Cys
D: Asp
E: Glu/Gln
F: Phe
G: Gly
H: His
I: Ile
K: Lys
KEGG: Kyoto Encyclopedia of Genes and Genomes
L: Leu
M: Met
N: Asn
NCBI: National Center for Biotechnology Information
NLSs: Nuclear localization signals
P: Pro
Q: Gln
R: Arg
S: Ser
T: Thr
V: Val
W: Trp
Y: Tyr
