# Supplementary material for: Evolutionary gradient of predicted nuclear localization signals (NLS)-bearing proteins in genomes of family Planctomycetaceae
Source: BMC Microbiol. 2017 Apr 4;17:86. doi: 10.1186/s12866-017-0981-y (PMC5381049; doi:10.1186/s12866-017-0981-y)
Supplement: Supplementary file 2 — Figure S1. Regulatory pathways of NLS-bearing proteins of Planctomycetaceae and fungi. Pathways colored pinkish red show NLS-bearing proteins of fungi; pathways colored green show NLS-bearing proteins of Planctomycetaceae; pathways colored light blue show the common Regulatory pathways between Planctomycetaceae and fungi. Dataset S1. The 13 clustered NLS-bearing protein families among non-planctomycete bacteria, Planctomycetaceae or fungi. Dataset S2. NES-bearing proteins in the predicted protein pools of the 27 strains. (PDF 408 kb) [file 12866_2017_981_MOESM2_ESM.pdf]

Supplementary Material Files - Supplementary Figure and Datasets

Title: Transformations of predicted nuclear localization signals in genomes of Planctomycetaceae species

Authors: Min Guo, Ruifu Yang, Chen Huang, Qiwen Liao, Guangyi Fan, Chenghang Sun, Simon Ming-Yuen Lee

Author for correspondence:

Simon Ming-Yuen Lee, State Key Laboratory of Quality Research of Chinese Medicine and Institute of Chinese Medical Sciences, University of Macau, Macao, China, Phone: (853) 8822-4695, Fax: (853) 8822-1358, simonlee@umac.mo.

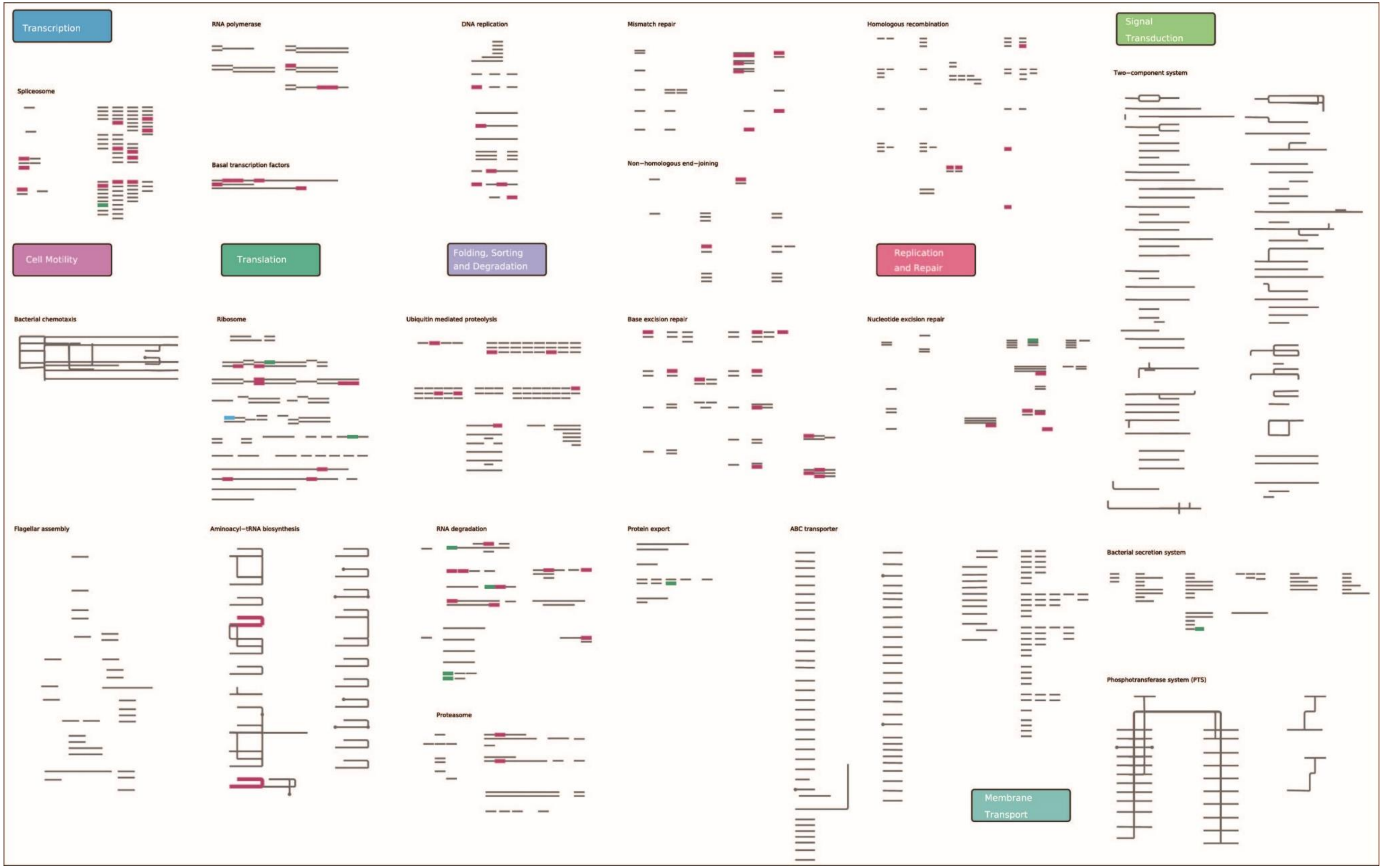
Transcription
Spliceosome
RNA polymerase
Basal transcription factors
DNA replication
Mismatch repair
Non−homologous end−joining
Homologous recombination
Signal Transduction
Two−component system
Cell Motility
Translation
Folding, Sorting and Degradation
Replication and Repair
Bacterial chemotaxis
Ribosome
Ubiquitin mediated proteolysis
Base excision repair
Nucleotide excision repair
Flagellar assembly
Aminoacyl−tRNA biosynthesis
RNA degradation
Proteasome
Protein export
ABC transporter
Membrane Transport
Bacterial secretion system
Phosphotransferase system (PTS)

Figure S1. Regulatory pathways of NLS-bearing proteins of Planctomycetaceae and fungi. Pathways colored pinkish red show NLS-bearing proteins of fungi; pathways colored green show NLS-bearing proteins of Planctomycetaceae; pathways colored light blue show the common Regulatory pathways between Planctomycetaceae and fungi.

Dataset S1. The 13 clustered NLS-bearing protein families among non-planctomycete bacteria, Planctomycetaceae or fungi.
Note:
pro: prokaryote(Bacteria and Archeae), euk: fungi(Eukaryon), int: Planctomycetaceae

---

euk+int:

ORTHOMCL5
>Eremothecium_cymbalariaeGL01760_euk gi363751242refXP_003645838.1
hypothetical protein Ecym_3547 [Eremothecium cymbalariae DBVPG#]
-K[RK]{3,5}.{11,18}[RK]K.{2,3}K
MTLFKFDEEISKFTSNGSCVILPDELDDTQSNNSHHQEYGSYVDEPSQELPWYKNLMSANPTALPSKNEPSTSPI
FHPGTSSNNKGNTTSTRIRRRSSLYNKFPINTPPNTTRFSLSVEEEPAIQEFYRSSPDFKTKHAPRKLEDFKPIR
VLGKGAYGKVLLVKDQLTNKLYAIKQLKKAEILITQQEGIDEYADEDNTINTTIKKRVERTFAERSILSQLEHPN
IVKLFYSFHDHHRLYLLLQYIPGGELFYHLKEQGTLDETTVAFYAAELSCALKFLHGKGIVYRDLKPENCLLNER
GHLVLTDFGLSKKSVHQPDYPISGESVNELYSIIGTPEYCAPEILEGLPYTQNCDWYSLGSLAYDMLIGKPPFTG
INHKVILSKIQREKSVKLPSYLSDGMKDYLNALLKKDTLKRWDVDIFWNKEGPKTKKKKAGQTKTTAYQSHFIFR
KINWKLMENGELQKTTVGPILPVITDWELAENFDSEFTERRLDSEDFQANGIAIQDHRTKYFNEPNKNDIFKGFS
FVASRSYLDRYF
>Eremothecium_cymbalariaeGL02413_euk gi363752549refXP_003646491.1
hypothetical protein Ecym_4652 [Eremothecium cymbalariae DBVPG#]
-[KR]{4}.{20,24}K{1,4}.K
MLDVEQDILRKIEREKNIIQGASNLKKRTDNVMVIQKCNTNIREAQQNIQYLEETLNKLQLTEDPVQTLSVHPGY
GQLSTIAPHEHRFSRLDLSKYDCPSLSQRIQYMLQQLEFKLHVEKQYQEANDKLTKLYQIDGDERSSSAAQGGAQ
ESKQRIQLLTKALKKYQAINVDIDQFRDSNDDLLAVQPKFRRKQLTGEFTLGISAIRDVDHIQSPLFSKNPETII
TVKIDDVVRAKTKPTRTDRWHEEFNLYVDKGNEVEITVYDRINDRIVPVAIMWLLLSDVTEEIRKKKVGQVGSPN
WFDVANPLTDQDPYVNTSTAHLPLGGLASASGLLGTADLSSETAPLKQPITTNAWFVLEPAGQIMLTLGFNKSNQ
NERKDLMGALRRHGAIVNRRDEVYEKHGHHFGQKSFYNIMCCAYCGEFIRYSGFQCQDCKFLCHKKCYNNVVTKC
IAKTSTDTDPDEAKLNHRIPHRFEPVSNRGTKWCCHCGYILSWGKKNVRKCSECGIMCHTQCAHLVPDFCGMSME
MANKVLTAIQDTKRSQQQRLRVSPSSNGSDEPAMMRADMSNTAASTKSPVTSQRNTLNSELSAESQTLDGQRYSN
YQATSSSQRSSNLPNDYEDTASKEKFINSPPDFADQSDLTDYTTYYKYTDMSSNRFSLSDQPSTLDPSSGSPQDG
GKQGEEDKTLLADEKSNLYEENLDEELRIDELKKTLEQEEVALMDSHRDLFIPEQSEDNAERKSFRLSDGITDLL
DLPMTVGTSPIEFTGISTDLSTDQSQNDDIVVTPIQPLSTSPIKPHSAGRHKRKSPKRRKVSLDDFILLKVLGKG
NFGKVLLAKSKNTDRLCAIKVLKKDHIIQNHDIESARAEKKVFLLATKAKHPFLTNLYCSFQTENRIYFAMEFIG
GGDLMWHVQNQRLSVRRAKFYAAEVLLALKYFHDNGIIYRDLKLENILLTLEGHIKIADYGLCKDNMWHGNKTST
FCGTPEFMAPEILKEQAYTKAVDWWAFGVLLYQMLLCQSPFSGDDEDEVFNAILTDEPLYPIDMAGDIVQIFQGL
LTKDPEKRLGAGTRDALEVMEEPFFRNINFDDIFNLRVESPYIPIIKAADDTSYFEKEFTAAPPTLTPLPSILSS
NLQEEFRGFSFMPDDLVL
>Saccharomyces_cerevisiaeGL00101_euk gi330443395refNP_009445.2 Pkc1p
[Saccharomyces cerevisiae] -[KR]{4}.{20,24}K{1,4}.K
MSFSQLEQNIKKKIAVEENIIRGASALKKKTSNVMVIQKCNTNIREARQNLEYLEDSLKKLRLKTAQQSQGENGS
EDNERCNSKEYGFLSTKSPNEHIFSRLDLVKYDCPSLAQRIQYMLQQLEFKLQVEKQYQEANTKLTKLYQIDGDQ
RSSSAAEGGAMESKYRIQMLNKALKKYQAINVDFDQFKHQPNDIMDNQQPKFRRKQLTGVLTIGITAARDVDHIQ

SPMFARKPESYVTIKIDDTIKARTKPSRNDRWSEDFQIPVEKGNEIEITVYDKVNDSLIPVAIMWLLLSDIAEEI
RKKKAGQTNEQQGWVNASNINGGSSLASEEGSTLTSTYSNSAIQSTSAKNVQGENTSTSQISTNSWFVLEPSGQI
LLTLGFHKSSQIERKQLMGGLHRHGAIINRKEEIFEQHGHHFVQKSFYNIMCCAYCGDFLRYTGFQCQDCKFLCH
KKCYTNVVTKCIAKTSTDTDPDEAKLNHRIPHRFLPTSNRGTKWCCHCGYILPWGRHKVRKCSECGIMCHAQCAH
LVPDFCGMSMEMANKILKTIQDTKRNQEKKKRTVPSAQLGSSIGTANGSDLSPSKLAERANAPLPPQPRKHDKTP
SPQKVGRDSPTKQHDPIIDKRISLQTHGREKLNKFIDENEAYLNFTEGAQQTAEFSSPEKTLDPTSNRRSLGLTD
LSIEHSQTWESKDDLMRDELELWKAQREEMELEIKQDSGEIQEDLEVDHIDLETKQKLDWENKNDFREADLTIDS
THTNPFRDMNSETFQIEQDHASKEVLQETVSLAPTSTHASRTTDQQSPQKSQTSTSAKHKKRAAKRRKVSLDNFV
LLKVLGKGNFGKVILSKSKNTDRLCAIKVLKKDNIIQNHDIESARAEKKVFLLATKTKHPFLTNLYCSFQTENRI
YFAMEFIGGGDLMWHVQNQRLSVRRAKFYAAEVLLALKYFHDNGVIYRDLKLENILLTPEGHIKIADYGLCKDEM
WYGNRTSTFCGTPEFMAPEILKEQEYTKAVDWWAFGVLLYQMLLCQSPFSGDDEDEVFNAILTDEPLYPIDMAGE
IVQIFQGLLTKDPEKRLGAGPRDADEVMEEPFFRNINFDDILNLRVKPPYIPEIKSPEDTSYFEQEFTSAPPTLT
PLPSVLTTSQQEEFRGFSFMPDDLDL
>Saccharomyces_cerevisiaeGL00232_euk gi6319502refNP_009584.1 Ypk3p
[Saccharomyces cerevisiae] -K[RK]{3,5}.{11,18}[RK]K.{2,3}K
MIFSLDEELHRVSLDDKKNDIKVDYSSAIYNDINHEQGSSITYEESINHLSVHSNAIPLNGMSPAHRMRRRSSAY
SKFPILTPPNTRRFSITGSDAMRTNTNRLSITPQDIISSNIGENELSRNLHDFKPVRVLGQGAYGKVLLVKDVNT
SKLYAMKQLRKAEILISQTATDSKREDEDKNDGNNNDNDDGLSKRLERTFAERSILSEIEHPNIVKLFYSFHDNS
KLYLLLQYIPGGELFYHLKEHGTLDETTVSFYAAEISCALRFLHTKGVVYRDLKPENCLLNQRGHLVLTDFGLSK
KSANDSAVDEEDPENVNALYSIIGTPEYCAPEILLGKAYSQNCDWYSLGCLLYDMLVGKPPYTGSNHKVIINKIQ
QNKQGPKIPFYLSEGMKDILNALLKKETAKRWNVDKYWAKTGANNKPTKSKKKKSGAARTSLFTEHFIFRKIDWK
LLESGQLQKTTLGPIVPVITDLELAENFDTEFTSMSYEETYTDSKPININSVSKSPDMFKGFSYKASGSYLEKYF
>Gemmata_obscuriglobusGL06171_int gi168704041refZP_02736318.1 probable
serine/threonine-protein kinase pknB [Gemmata obscuriglobus UQM 2246] -RPRRK
MSDPNDPSLIIDSRSGSLLISTLDHLGATKFTTPRGSAAAPTFSRPATEDELGTLGPYRVVRELGRGAMGAVYLA
RDTRLDRSLALKVMLPEFAADAEAKERFLREAKSAARVSHDHVVTVYEADERDGTPYIAMQLLQGYSLDAFIRNK
GTPSLRNAVRIAREAALGLAAAHKLGIVHRDIKPANMWLEAPRGRVKLLDFGLARPVAMETELTQSGAVVGTPAF
MSPEQALGERVDHRADLFSLGAVLYLLCTKQLPFPGASLSAVLLALGTKEPTPVHAHSPNVPEPLADLIHGLLAK
DPGNRPQTAVTVANRLHQISEHLAGLAAAAPPEGPDSPPPRPDAQSTDPWATRIPSSSAHPSGKRAAPPGSARAI
RSMPVVILSPTPRPEGPRESEPSRESQKTPDALHTVISSGVVWKEEPDAGKAEKESRERPRRKRAKSKSVSVAPI
LVAAGSVALFAVLVAIMLTAAGGKPRATDSARRETNTGLPPAATGNETLPPIVREGNVAKGGGSTGSIALEGKAP
MRDSSASGATVDLLALVDTRVHTVLGEWRRNGEALVGVNPQFPGVLQLPYEPGEEYDIEATVRRISGDEYFGFQL
VAGGHRVNMAIDTWPSKGYFSGMGSIAGKDLLNNGTGTQGRRLVHAGTAYTITSSVRKGRISTSVNGILVTSYTG
EFNQFSIHSDFRVQNPKALAVQIGYATPFQIDRLVVTPVSGNGKVVH
>Gemmata_obscuriglobusGL06513_int gi168704383refZP_02736660.1
serine/threonine-protein kinase [Gemmata obscuriglobus UQM 2246] -RKRRR
MPDDLSATRVSGPNSPHAPDSSATVARFTDPLAARRQMLGEEVPAVPGYEVARELARGGMGRVLAARELTLNRPV
AIKVLLPRAGRDGARRFVIEAEITAQLPHPNIPPVYALGRFGDGTPFLVMKLIEGRTLTALLRDRASPADELNRF
VQIFEHVCLAVGFAHARGVVHRDLKPANIMVGEFGEVQVMDWGLAKTAGGPDAPTRAQGTGDEPKTLGDRTGSSD
AFSSDHSTEVTAAGQVLGTPSYMAPEQARGEPVDPRADVFSLGAVLCEVLTGSRPFAGRHSTEVIGATAAGILDE
PLAALGASGAPADLVELARRCLAPWAADRPADGKAVADAVARYRAGVETRLRAAESERAAAEVKATEQRKRRRVQ
LALLAAVALMLLGAAGTGWWLDHKAAERRIEAERAERERAAADLRQSEKDARAAADNRRALAADLDRCEAALRND
DAATAALALADVTRREGQPGGEEFGARIARCRTDHALLTRLDEVETFAWTPVNGQLPLAADLQTRWATALAQAGI
VPNAPPAEAARRVNDSLIRDRVLLTLDLWLGAAGTADLAALLHEADPDPTRDAVRAAVQARDGAGLARLTAAGLG

DQPARFVIALAAAVRPGLSPARRRALLAAEWDRAPGSVRVLMELGGTYPMNSKEGAAERARWFQAAVALRPQSVL
AWNKLGIALRDGEQYDEALAAAREALRLDPRDLNALNIKAIALRAKGDGPGAVRVYEELMRADPDCPYPEFHNNY
GSALATAGDLRGAIVKFRQALEMNPKFAAAHANLGNALLRTRELDAAVQSLRAAVECDPRSAVAHAELGRALQLK
RDTTGAIRAYRESLKLNPANPGAASALGHLLWDREDLDGAIGAFRDALRYAPAEPGLHNNLGMLLRQTGDPRGAA
SAYKEAIRLDPKHLRARYNLGVALRAAKDPAGAVKAYRELLALDPNHSAGHNGLGNALRDLGDLDGAIAEFRAAL
SINPSNGPAQQNLTDTLKQKDRTAPRPREVKRS
>Isosphaera_pallidaGL02683_int gi320104238refYP_004179829.1 serine/threonine protein kinase [Isosphaera pallida ATCC 43644] -RKRRR
MSQQFNPTGSRWPWQYPPRNSEPPLLDSQPTAPSLKPSSSCERSFRQTIAPGMRVGRYLIEEPLARGGMGFVLKA
FDLEMERRVALKVLNDSLASNPDLRQRFVNEAKITGRLEHPSIPPVYELSVWGDGRPFLAMKLIQGRTLSELLTS
RVGSSDELPKFLSIYLKICEGLAHAHRRGILHRDLKPSNIMVGDFGEVQVMDWGLAKDLNEWSRRRVPRARELTK
STLLDVAMMVDSSPESWEGMGTPAYMAPEQADGDPHHLTPAADVFGLGGLLCVILTGRPPYVAASRAELLRKAAM
ADLDDAFALLEQAPVEPQWKDLCRRCLDPQPLKRPANAVEVAQLVSSLLDETEARARRAELDAAVQMTQLAEERK
RRRIGFLLALAILVGGFVGLEWLRQRQAQRETTWTTLQNRLIQAEEARQRRLWNLGLNELAEAQLLALSLEDHSV
IDQVQHLAEQLTLERDLEEIRLDRAQWGPEGFPKVEDRYAERLRRIGFDLVNPDDTVLAQILRTAPSFPRLSDAL
DDWIVEASRDEVRTRLLRIQHLATGESAYFRLAELDWSSKISLQALYRILEEIPPDKLGLIQLIAYALDRAGGDA
TRVLEQAILRHPEEFWLHLHLGNILSREQSHTDSLERAVRAFGTARAIRPDNFAAINNLALALRKLKRFDEALIL
HRKALQISPDLALGRLNLATCLSELGHHAEAENLLRSALSLEPDNTLLLYSLSRVLFRQKRSAEVLEIARREAEL
QPSSWRPLLLQSHLLREMGQDDEALTVTERAARLAPLETDVQLELARIFAKRQELDRAVHHLQCALATEPISPRL
HLDSRAILESIHRVEEAVEHQRIMVALEPDSWENLADLAAMLKKLDPGMKVFGAEIEAVLLRATQLAPRQRRPYL
MLASFSTQARKHAQALEMFEKALALDPDDLETRSDLAITLVMLGRRSEAMAIWHQLSEQAPDDFRFHANLGAALA
EEGQVDQAIVAIERAIQAFDRAGDSPNARPTQFPRDVARANLTKMLQALRARRQP
>Planctomyces_limnophilusGL00813_int gi296121087refYP_003628865.1 erine/threonine protein kinase-related protein [Planctomyces limnophilus DSM 3776] -P.KKKRK
MAGRMIGPFELGDRLGVGGMGIVYRATYTKTGAPVAIKILSPDLSQAESLQKRFEREVAILKKLQHPHIVRYYGG
GKLGTQRFYAMELVTGGSIEGYLKEHGQLPWKEVLDLALQIAQALEHSHAAGVIHRDLKPANLLRAKDGTLKLTD
FGIARDTTATALTAAGRTVGTYSYMAPEQIRGKPPVDRRTDLYALGCVMFEMITGETPFRGDNAGEMLIMHLQED
PPRPSSLNAECPQVVEDLILKLLAKDPEDRFFDALAVQVAIEQIQEQLTKPVEAISPEAAKTAEANVPKKKKRKK
SAKVPFYERAWFLASILVTILFLATIPFWPMSEDQLFARAEALMKSEEPTDWGDAYDKYLKPLQQKFPQGKHAGQ
VQQYVDQVEMHQAERRARATAKTGRDPNSEAERLFLEADRYEKFGDRISALEKYESMVVLLKDREQDRAFVMLAK
KRIQEIEAAGGDPVDRVQMVDQSLARADELAQQGKTIEARTIWNSIMTLYAGNQELEVQVRQARQRLSEKRSSSE

ORTHOMCL52
>Eremothecium_cymbalariaeGL00971_euk gi363749663refXP_003645049.1 hypothetical protein Ecym_2511 [Eremothecium cymbalariae DBVPG#] -[KR]{4}.{20,24}K{1,4}.K
MFLLRLSTWSSKLLPRIVMSPASQPVSTRFFSVSPVSFATLNQIKRGVQPPRRKKETESPDLERCPIRKGVVLRV
MVLKPKKPNSAQRKACRVRLTNGNVISAYIPGEGHNAQEHSIVYVRGGRCQDLPGVKYHVVRGAGDLAGVVNRIS
SRSKYGVKRPQKE
>Saccharomyces_cerevisiaeGL04823_euk gi6324364refNP_014434.1 Mrp12p [Saccharomyces cerevisiae] -[KR]{4}.{20,24}K{1,4}.K
MLSRFMSNTWCTPLRQAQRLFSSTTTMQATLNQIKRGSGPPRRKKISTAPQLDQCPQRKGVVLRVMVLKPKKPNS
AQRKACRVRLTNGNVVSAYIPGEGHDAQEHSIVYVRGGRCQDLPGVKYHVIRGAGDLSGVVNRISSRSKYGAKKP

SKS
>Blastopirellula_marinaGL05242_int gi87306543refZP_01088690.1 30S ribosomal protein S12 [Blastopirellula marina DSM 3645] -P.KKKRK
MPTINQLIRRPRKKKRKFSKSPVLEKCPQKRGVCLQVRTMTPKKPNSALRKICRVRLSNGKEVTVYIPGEGHSLQ
EHSIVLVRGGRVRDLPGVRYQVVRGSLDALGVNGRKQSRSRYGAKKS

ORTHOMCL86
>Aspergillus_nigerGL02657_euk gi317028624refXP_001390395.2 polyketide synthase [Aspergillus niger CBS] -RKRRR
MVAFSGKRAHDAPGENILAPLAVVGMSLKFPEDATSPEAFWKMLVEGRCVSTEFPSNRMNIDAHHDAERGRLHSI
SCRGAHFLKEDLGLFDAPFFGITDTDAKAMDPQQRLALETVYRALENAGLPIEQVAGSKTSVFAGSFCSDYHMLQ
IKDPLNVPKNATAGTGRNMIANRISWFYDFLGPSATIDTACSSSLMAVDLACQSIWGGDAAMGVAIGCNIILAPE
MTIGLDNLGLLSRDSHSYSFDKRANGYARGEGVGAVVIKRLDDAIADGDSVRAVIRSSSSNQDGRTPGILQPSKD
AQVRLIRDTYQKAGLDMGVTRYFEAHGTGTPIGDPIEARAIGTAFRSYRSEQAPLHVGSVKSNIGHLEGASGIAG
FIKAVLVLEKGFIPPNSNNLQHINPQIDEDYLRLKILNKAIVWPTTGIRRASVSSFGFGGANSHIVLDDAYNSLQ
LGGFEQVGHQTVRVPSLGQVNGTPQLNGHHDTGGTDTFNDSTIACKTVPKLVVWSAADHAGIKRLAESWSTYLSD
LSVEETEEYLRDLAHTLCDRRSHWAWRTFVVAKPGVLLQDLTRQFSPATQSIDSPHLAFVFTGQGAQWYAMGREL
IGHYEAFTRSLTESGSYFKELGCTWDILEELQKPGLDSNVNDPSYGQPLCTALQIALVDLLESWGVSPAAVVGHS
SGEIAAAYSSGALTKWSALKVAYFRGSLAGVLGRSSSIKGAMLAVGLSRENTQKYLDALEPQFERVEAVVACVNS
PQSVTISGKLEQIDALHDLLDRDGVFSRKLAVNVAYHSFQMREISDRYFTALGDLEAPCKRKRRRPFMVSSVTGT
LVSSERLVEPEYWVTNMVSPVLFHDAISYLCSQSGKTYKKIDGSHRHAITINHLLEIGPHCALQGPCRDIVSVLK
KSDRVTYIPLMIRNRSALECAMEAAGRLHCSGYPIKLALVNDNGEAKTQRRPRVLVDLPEYPFNHSTSYWHEGRL
SEGYRFRRYGYLELLGTPEPNGNPMEASWRNIIRVSDMAWVQDHKINNTILYPGAGMLVMAIEAVKQLAEPDRLV
IGFIIRDAVFSTALQIPTRGRSDATGWYEWRIYSYDNGNWVENSTGSIQALYESRNTGLDANIREERAWESHLLE
TYNTVVRSCTSTVDAKSFYKHLNSCGYQYGPEFAAIKSIGYSETDCKALITGIRTFQPTGVYPDHTIHPTTLDAI
IQMAAGLESNMGQSVASVAVPVRIDRLWLSNTGGLSHPLADAVRAYAACSQSAVGYKSYSMTAVDGEVSKALLNL
EGLKVTAIAGSETTPVSDPFTKDNLCHYIAHKPDIDLLTIEEAQRLYGAHESQVIEPVEHFTELDFVAAASVSRY
AASFREEERDNIPPHLNKYIDWALEVKRTVDQGLSEFSAKEWIDRMGDDDYVSELHRRVESGSKRGQLTSTVCRN
LPDFVKDPLTHLFGNNLLAEVYREMLYADSIKPRLDRVIGALGHKNPRMKILEVGAGTGAMTDFCIRALSMDTST
DASLRRYGQWDFTDISSSFFPGAQEMFAAEGQRMRFKVLDIEKDPEIQGFECGTYDMVVAFMVIHATKDLAVSLR
NVRKLLKEGGKLLLFEITHFHPLRVNLIFGLLDGWWRSTETYRQTSPCISSEKWGELLKETGFSGCDLVVDDYDA
DICREGSMIVSTAVAPSPVEITTVNIIIQTEDQTQADLAAAISGRLQQLGISKITLTSLTDMAQRKLSTDLLDIS
LLESTTPFICDMDSSEYEGLQALVASTKSLIWVGEGGGRQPHPKYRLVDGLFRVLSGEMYRARLTNLSLERHSSR
KHKAAQICKLVFSSIGDMDRSADTEYTEIDGVLHVSRLVDARSLSQGVVRKALPQQEGLLPYGSGPSLRLSIGSP
GLLNTLHFVEDRSLQKQLGPRDIKIKVKAVGLNFRDVLVALGRLESDTLGAEFAGEVVQVGDQCQKFQPGDRVVA
FHASRYANYVTVREDMPVVGIRNEKMLFTTAAAIPVAYATAWITLTKIAGLQAGESILIHSGAGGTGQAAIQVAR
YLGATVFATVSTDEKRQLLMDRYNIPTEHIFSSRNTLFAKGIRRLTTDRGVDVVLNSLSGDGLIASWECIAPYGR
FVEIGKNDILSNSKLPMLQFERNVSFTAIDLAEMAIDRPHIIRAALETVFSLLEEGKLDLVYPLQIRGIADIEQA
FRQMQTGKNSGKTVLEMRETDQVMTVLDTKPSYTFGPDATYVIAGGLGGLGRSIARWLVERGARNLILLSRSGPE
SPHARSLVEELHERGARAITPACDITNRELLKTVLDVCSQLMPPIKGCVQASMVVRAHNFESLPYQSWKETTAPK
VQGSWYLHELLPRGMDFFVLMSSVSGIMGVVSDSGYAAGNTFEDGLARYRVGLGEKAVSLDLGLFLTAGYLKENP
ESREQFLANTVLDEIQESHLHALLDTYCDSTQGPISMQESQVVVGITPSRQKLETRKAEWLDRPLFRHLSLTDGR
TEGRGSSEDSNLAALFAGASSTEEAAAIAMRATREKLSIMMSTPVDEIDTDKPFHQYGVDSLAGVELRNWFAREL
RADLAMFDILGGASLASVVTLAVGKSEYRR

>Schlesneria_paludicolaGL00043_int Planctomyces limnophilusGL000043 locus=Scaffold14717954270- -KRPRP
MSMTDRTNRIAIVGVGGIFPGAHELDQFWQNIANGVDSGRSAPRERWFLAPETAYDPVGPKPDRVYSTWGCFIEG
FEFDPSGLDLDHQLVRQLDPLFHLGLHAARAAVQDARSLDGIDRRRVGVILGNIALPTESTSAICRDVLGRTLIE
KLIDSVPRSDARRAQLQQELAEGYASKSWHPLNHFAASLPAAMIAQGLGLGGSAYTLDAACASSLYALKFACEEL
RDHRADAMLAGGMSRPDCQYTQMGFAQLKALSRGSRCAPLSSSADGLVVGEGAGVFVLKRLNDAIAARDRIYGVI
AGIGLSNDRGANLLAPHSEGQLRAMRAAYQEAGWRPSDVDLIECHATGTPVGDGVEIASLHQLWQDESSRSGQCV
IGGVKSNVGHLLTGAGAAGLMKVLMAIQHQTLPPTANFTTPAAPLSDPKSPFRVLGQSERWPRRGNGRLSARSSL
HDNARSEVPRRATVNAFGFGGINAHVLIEEFLDSAIALSVPSQPPPISLDAAVVERRTTSCDESTIEIAIVGLAV
QVGESDAGFVECLEPASNLTPDSAAGTQDWGVLESEWFRKEFGDQRKHFEGLGKIRSVTTPLGEFRIPPLELQDM
LPQQQLMLKLASSALHDVSPAMATSVESVSDRTGVFIGIELDPNTSGFHLRWSIEQVAPRWAEQLGLLLSAAELR
AWTDNLKDRVSPPLSANRVMGNLGGIVASRLAREFNVGGPSFTISSDQNSGFRALELAANALRRGELDQAIVGAV
DLPCDIRSQIARLRAVLADSQQPDHDGAVAVVLKRRDDAERDGNQIHAILSRIESVGWNVEPTRHRRLASLGAAT
GLVNFVQAVQAIEVPLRETPSKQYWLTNRDDGPRTMQIPGCGVDGQALRFELMGSAKRSATLAGNSTSDAFGIGV
FVMDGDSTAEIATSLDELKDQVQAAGQSPQGGQVRSQMASSMGRLAQAWHRRRWSSVPRRLALVLLATSLDELAT
LIDVGSARLRGEAVAPEFDRRVHFSTRPLGPSGKLAFVFPGSGNAFLGMGRELLAAFPDVLRRQEQENARLRDQY
KSELIWNGSSTAALLDDHKSMIFGQVALGTAICDLLHLFGISPRASIGYSLGESAAFFGLRAWTHRDEMLQRMDE
ATLFGSDLVRPFRAARLAWNVPEAVEVDWISGAIDRGPDEVRDAISRLAPGGAASGRHVESGESSSSKPPARNAY
LLIINTPTQCVIGGQRVAVESLVRMLNANFVPFAAPSTVHCDILRQVESAYHALHDMPTTPPPVEFYSAAWGHAY
ELTRESATRAIVAQAVDTIDFPRVVEQAYADGVRIFVEIGPGSSCARMIDEILGDRIHLAQSATPITINPIVTFF
ELLARLIAERVPVDLSYLYGQVGRDTVEDQGRDKRREIVTRTSAAAFSVPVPTGGWPNEPVDSAVDDDGMAEMVE
TQAKNVGEASQPLFPHQDTDEILDMLGTNVITQLQEILAARQKAHETYLRVSGEIYQTTLSQLAALTELANLDGR
LSPSVSAPIADDRLPTEQVSPAILAVEPNAPTSDEPPRSLTREQCMEFAIGKIGRALGPMFAEIDQHPTRVRLPD
EPLMLVDRILEIEGEPLSMTSGRVVTEHDIHRGAWYLDCDRIPTCIAVEAGQADLFLSGWLGIDRETKGLACYRL
LDAVVTFHQALPRPGQTIHYDIKVNHFFRQGKTFLFRFEFDATVDGEPLLTMREGCAGFFSEQELASGKGIIHTA
LDKRPRPGKRPDDWVELVPLAIESYDDAQILSLRQGRLDQCFGDAFAGLALQTPVTLPGLPETSESLRSPQANVA
MATQAGAQRTRMWLIDRVLSLDPTAGKFGLGTILGELDIHSDDWFLTCHFCDDQVMPGTLMYECCMHTLRIYLLR
MGWIGEQGQVAYEPVPGIRSRLKCRGQVRADTKKVWYEITLKEIGYLPSVDGGLEQTPYCLADALMYADGKPVVE
ITDMSVRLTGLTRTHVERLWSQKKQVSGSLHGAGVTSSTVVKPRYDRRPALFDTDRITAFAIGNPSEAFGDRYRV
FDSERTIARLPGPPFQFLDRIVAISGCEPWVLRAGGEVVAQYDVPVDAWYFAANRQHQMPFAVLLETALQPCGWL
AAYVGSALTSDIDVSFRNLGGTATQFREVVPTSGTLTTTVKMTRVSNSGGMIIQHYDFDLRCDDQTVYVGNTYFG
FFSKASLANQVGFRDAKPFVPTPADLVHSIKFPYTDTAPFADRRFQMIDDVQLSQSGGPGGLGFAIGTTHVDPTS
WFFKAHFHQDPVVPGSLGLESFYQLLKFYAADRWKLSPNASFATPIIEGTSTSVPAKHEWVYRGQVVPRDQKVTV
SAVITRVDDQKQQLQAEGFLSVDGRIIYQMKQFSLGCS

----

pro+int

ORTHOMCL53

>Escherichia_coliGL02648_pro gi218696128refYP_002403795.1 polyphosphate kinase [Escherichia coli 55989] -PRRRK

MGQEKLYIEKELSWLSFNERVLQEAADKSNPLIERMRFLGIYSNNLDEFYKVRFAELKRRIIISEEQGSNSHSRH
LLGKIQSRVLKADQEFDGLYNELLLEMARNQIFLINERQLSVNQQNWLRHYFKQYLRQHITPILINPDTDLVQFL
KDDYTYLAVEIIRGDTIRYALLEIPSDKVPRFVNLPPEAPRRRKPMILLDNILRYCLDDIFKGFFDYDALNAYSM
KMTRDAEYDLVHEMEASLMELMSSSLKQRLTAEPVRFVYQRDMPNALVEVLREKLTISRYDSIVPGGRYHNFKDF
INFPNVGKANLVNKPLPRLRHIWFDKAQFRNGFDAIRERDVLLYYPYHTFEHVLELLRQASFDPSVLAIKINIYR
VAKDSRIIDSMIHAAHNGKKVTVVVELQARFDEEANIHWAKRLTEAGVHVIFSAPGLKIHAKLFLISRKENGEVV
RYAHIGTGNFNEKTARLYTDYSLLTADARITNEVRRVFNFIENPYRPVTFDYLMVSPQNSRRLLYEMVDREIANA
QQGLPSGITLKLNNLVDKGLVDRLYAASSSGVPVNLLVRGMCSLIPNLEGISDNIRAISIVDRYLEHDRVYIFEN
GGDKKVYLSSADWMTRNIDYRIEVATPLLDPRLKQRVLDIIDILFSDTVKARYIDKELSNRYVPRGNRRKVRAQL
AIYDYIKSLEQPE
>Escherichia_coli_O157GL03252_pro gi15832617refNP_311390.1 polyphosphate
kinase [Escherichia coli O157H7 tr. Sakai] -PRRRK
MGQEKLYIEKELSWLSFNERVLQEAADKSNPLIERMRFLGIYSNNLDEFYKVRFAELKRRIIISEEQGSNSHSRH
LLGKIQSRVLKADQEFDGLYNELLLEMARNQIFLINERQLSVNQQNWLRHYFKQYLRQHITPILINPDTDLVQFL
KDDYTYLAVEIIRGDTIRYALLEIPSDKVPRFVNLPPEAPRRRKPMILLDNILRYCLDDIFKGFFDYDALNAYSM
KMTRDAEYDLVHEMEASLMELMSSSLKQRLTAEPVRFVYQRDMPNALVEVLREKLTISRYDSIVPGGRYHNFKDF
INFPNVGKANLVNKPLPRLRHIWFDKAQFRNGFDAIRERDVLLYYPYHTFEHVLELLRQASFDPSVLAIKINIYR
VAKDSRIIDSMIHAAHNGKKVTVVVELQARFDEEANIHWAKRLTEAGVHVIFSAPGLKIHAKLFLISRKENGEVV
RYAHIGTGNFNEKTARLYTDYSLLTADARITNEVRRVFNFIENPYRPVTFDYLMVSPQNSRRLLYEMVDREIANA
QQGLPSGITLKLNNLVDKGLVDRLYAASSSGVPVNLLVRGMCSLIPNLEGISDNIRAISIVDRYLEHDRVYIFEN
GGDKKVYLSSADWMTRNIDYRIEVATPLLDPRLKQRVLDIIDILFSDTVKARYIDKELSNRYVPRGNRRKVRAQL
AIYDYIKSLEQPE
>Zavarzinella_formosaGL04702_int Gemmata obscuriglobusGL006710
locus=Scaffold4388921391104- -RKRRR
MSSTPQLSVAAPAPSINLDDPSLYINRELSWLEFNRRVLEEAQDKRVPFMERLKFLAIVSSNLDEFFMVRVGGLM
QKVHAGITRSSGADRTTPKVQLEKISQHISKMVQDQYHVLTEEVLPALEKEGIVIKSTVKQLTEADKKHLRENFR
REVFPVLTPLAIDPGHPFPHLANKTLNLAVVLERPSHPDKLYAVVQVPAVLRRFMPIPVEGGYCFASLEAVTRLH
LAELFPGMKIDHATVFRVTRDSEYEIEDEEVEDLLKAIEESVRKRRRGTAVRLEIEADAPEEVVNFLTSALDLDT
SADVFRVPGLIDLTGLFQVYGLPGFPHLRDPHFVPHPVPDIANAANIWAAIRTRDILMHHPYESFNPVVDFIETA
AADERVLAIKQTLYRTSSDSPVVRALQRAADAGKQVTAVIELKARLDEERNIAWARELEKSGVHVVFGFVGLKTH
CKVALVVRREEDGIRRYVHLGTGNYNPQTARLYTDLGMFTCNPDFADDVSALFNYLTGYSELPEWKKLIVAPSRM
QNFMVEMIDREAEFEKAGKGGRFIAKVNGILEPVIVQALYRASRAGMKSDIVCRGICALRPGIPGVSENIRVVSI
VDRYLEHSRIYYFGNGGDPVVYVGSADLMDRNLSRRVEVIFPIEQPDLKQRVIKEILEVTLSDTAKSRELQSDGS
YKRVEPSAGAKPVSSQSIFQANAADAKRDCPPMPPPGDVEEKPIRRNRKRNP

ORTHOMCL63
>Parachlamydia_acanthamoebaeGL00608_pro gi338174602refYP_004651412.1 hp70,
dnaK gene product [Parachlamydia acanthamoebae UV-7] -[KR]{4}.{20,24}K{1,4}.K
MNKKKGKIIGIDLGTTNSCVAVMEGGVPKVIASAEGSRTTPSVVAFKGNERLVGIPAKRQAVTNPENTIFSSKRF
IGHKYSEVVNEIKTVPYKVTENAKGDAVFEVQGKIVTPEEIAAQILIKMKETAEAYLGEKVTEAVITVPAYFNDS
QRQSTKDAGRIAGLDVKRIIPEPTAAALAYGLDKEKTEKKIAVFDLGGGTFDISILEIGEGVFEVLATNGDTHLG
GDDFDHAILNWMLETFKQETGIDLHNDKMALQRLRDAAEKAKIELSGTQSTEINQPFITMDATGPKHLSLNLTRA
KLESLTAELIDRTREPCIKALKDSGLSKDDIGEVILVGGMTRMPAVQEVVKSIFGKEGHKGVNPDEVVAVGAAIQ
GGVLAGDVKDVLLLDVTPLTLGIETMGGVMTPLVERNTTIPTQKKQVFSTAADNQPAVTIRVLQGERKMANDNKE

IGRFDLADIPPAPRGVPQIEVAFDIDADGILHVSAKDNSSGKEQKIRIEAQSGLREEDIQNMLKDAELHSEEDKK
RKEEVEIRNEADSQAFRASKALDEYKDKLPAEIVSEVQGKIDAVKKALEGTDSARIKSAKEDLEKSMQHIGEAMA
KAGAAGGAHASAAAHEGQAHQQSSSFEGGQSHGGHHHEHSKDDDQIEEAEVEIIDDKDK
>Isosphaera_pallidaGL00703_int gi320102258refYP_004177849.1 chaperone protein
DnaK [Isosphaera pallida ATCC 43644] -[KR]{4}.{20,24}K{1,4}.K
MAEGEKIIGIDLGTTNSVVAVLEGGEPVVIANQEGSRLTPSVVAFTTKGDSILVGEPAKRQAVTNPTGTIYSIKR
FMGRRHSEVEAEEKMVPYKVVGGPNDYVKVEAGGKTLTPPEVSAMVLRKLKEAAESYLGHKVRKAVITVPAYFND
AQRQATKDAGQIAGLEVMRIINEPTAAALAYGLDKKKNEKIAVFDLGGGTFDISILDVADGVFEVLSTSGDTHLG
GDDWDEALIDFIAEEFKREQGIDLRKDPMALQRLKEAAEKAKKDLSFQTQAEINLPFITADQTGPKHLMQTITRS
QFEKLTDHLFERCRRPVFKALEDAKLKPSDIDEVVLVGGSTRMPRVQQIVKDIFGKEPHKGVNPDEVVAIGAAIQ
GGVLTGDVKDLLLLDVTPLSLGVETKGGVFTVLVPRNTTIPTKKQEIFTTAEDGQTAVTIQVFQGERPMAADNRL
LGKFNLEDIPPARMGVPQIEVTFDIDANGILQVSAVEKGTGKQASVRIEASGGLSKEEIERMQREAEANAASDKK
RRELAEARNEADQAVYRIEKTLEDAKDKLTEADTQAVKAAIERVKNAVKGDSIEAIRQATEDLKKAGMAMSEHLY
ASNQAGSSSPVGAGATTGNSAHKEDETIDVEFEEKK
>Singulisphaera_acidiphilaGL01922_int Isosphaera pallidaGL002860
locus=Scaffold2214635216572- -[KR]{4}.{20,24}K{1,4}.K
MAEGEKIIGIDLGTTNSVVSVMEGGEPTVIPNQEGSRLTPSVVAFTSKGEVLVGEPARRQAITNPKGTIYSIKRF
MGRRHKEVATEEKMVPYAIVGGPDDFVKIQVGGKEYTPPEISAYILRKLKEAAESHLGHKVRKAVITVPAYFNDS
QRQATKDAGQIAGLEVARIINEPTAAALAYGLDKKKNEKIAVFDLGGGTYDISILDVGDGVFEVLSTHGDTHLGG
DDWDEALINDIANTFKKEQGVDLRKDQMALQRLKEAAEKAKKDLSFQAQADINLPFITADQNGPKHLTMTITRAQ
FEKLTDPLFERVKPPVRKALEDAKLKPAEIHEVVLVGGATRMPLVQQIVKELFGKDPHKGVNPDEVVAVGAAIQG
AVLTGEVKDLLLLDVTPLSLGLETKGGVFTKLVERNTTIPTEKKETFTTADDNQTAVTIKVYQGERPMAADNRLL
SEFNLEGIPPARMGTPQIEVAFNLDANGILQVSAKDKGTGKEQSVKVESSGGLTKDEIDRMQRDAAAHATEDKRR
RDLAEARNSAEQRVYQLEKLLEENKAKLSESDMAAVRSAITKVNQVKTGEDTAAIHQALEDLQRASQAMSEHLYA
APAAPGAEAASGAATSSNGGAQAGQGQKQGKGEEEVIDVEFEQKT

ORTHOMCL67
>gi76789128refYP_328214.1_pro exoribonuclease II [Chlamydia trachomatis
A/HAR-13] -K[RK]{3,5}.{11,18}[RK]K.{2,3}K |[KR]{4}.{20,24}K{1,4}.K
MGKAKNKKKFLKNRKQVLVPGTLFVHSRKGFGFVSPDQPELYPFDIFISASDLKGALDGDHVLVALPFSLRGGEK
RKGVIHKVLSRGKTVLVGTIVSLINPTLAMVCVNTIGPEHPLKAELLPKRTYKLGDRLLLKTPVWKENYPSKEPP
PLAMLEFIGNISNAKTDFPVIKAEFSITEEFPDAVVQEASQFLQKHVTQALHSRKDLRDLLCFTIDSSSAKDFDD
AVSLTYDHEGNYILGVHIADVSHYVTPNSALDREAAKRCNSIYFPGKVIPMLPSALSDNLCSLKPNVDRLAVSVF
MTFSKEGFLSDYRILRSVIRSKYRMTYDEVDEIIEKKQTHPISKTILKMAELSRIFSDIREQRGCTRLVLPSFTM
SLDNLQEPVALIENKQTAAHKLIEEFMLKANEVIAYHISHQGITMPFRTHEPPNEESLLVFQETAKAMGFTITQT
PAQEPDYQYLLQETTAGHPLEPILHSQFVRSMKTASYSTENKGHYGLCLDYYTHFTSPIRRYVDLIVHRLLFHPL
SVEEEHLEQIVRACSSQERIAAKAEGAFVNIKKARFLKKFIEEQPATLYKAFIITASPEGISFVLPEFCHEGFIP
AAKLPQAYVLQTKIGLEELPEYLRPGAVISVQLASVTLLTQSIEWTLVEATTKAKAKRTSKKKKTESVTTKEKKK
SPAKKKKGATKTKKGSGKN
>Pirellula_staleyiGL04627_int gi283782429refYP_003373184.1 ribonuclease R
[Pirellula staleyi DSM 6068] -K[RK]{3,5}.{11,18}[RK]K.{2,3}K
MNLPEFEEQSDRPLPESSSISDSSPLSELPESPADFPLEKDSPAARKLEPLVLAHVLAKNYQPVKPKVIAKQMKL
RSDQLPALKLAIRRLVKAGKLAYGSSHMVRKPDLLPPLPPESAKAREGTKKSKPARDLAVESTDDDLPDDVVNDL
ADETLDEAEAFAAEEAAADSSAEVDPDDFLAMRAAKLKEKPSRRGKDKTVTGKFKRAAAGFGFVRPLDVTSRGDR

TQDIFIPQNASQDAANGDIVRVRLVSGGRSLRKSGEIVEILERDTHQFVGVYQEQRGSGVVEVDGRVFAVPISVG
DPGAKGAAPGDKVVIEMVRFPSHTHEGEAVITEVLGARGTPGIDTLSIMREYELPEAFPEAVLAASREEAEKFDE
SIGDRRDFTKTTIITIDPVDARDFDDAISLTKLESGNYQLGVHIADVSHFVKPKSVLDREARDRATSVYLPDRVL
PMLPEIISNNLASLQPDKVRYAQTAIIEFSPEGTPLHSEFYLSAIKSCRRFTYEEVDDYLADRQAWKKKLKPQVH
ELLGVMHELAMILRRRRLERGAIELSLPETKIDLDKKGEVSGAHLSKNTESHQIIEEFMLAANEAVARHLNEKEL
PFLRRIHENPDLRKLQVLTKFVRELGIECDSLESRFEIKRVIKEVAGAPEEHAVNYAVLRAMQKAIYSPAVEGHY
ALHSEHYCHFTSPIRRYPDLTIHRMLRSLIHGKKPAADFASQVLLAEHCSEREQRAQAAERDLIKVKLLGYLSKR
VGSEMDAVITGVEEFGIFVMGIDLPAEGLIHIQSLEDDYYKFDSDTHSLTGRRAGNRFRLGDVLRVSVAKVDVDR
RELDFRFVGRLKSDRTHLSRLPRSGVRMRQDVPDEMQNDADFPPRRDEGSRPEFPDRGPPREGGARGGYQGGGSR
GKSFGSRSGGGRSSGGGDRSGGSFGSGGKKKKFPGSKPSFGGGGGKGKKGRKKGR
>Rhodopirellula_balticaGL04684_int gi32475350refNP_868344.1 ribonuclease R [Rhodopirellula baltica SH 1] -[KR]{4}.{20,24}K{1,4}.K
MQVSQELIDRVLRLVHAAEYRPSKPKQIAALLELDADGYREVRRVIKQLVLEGRLIYGGNHLVVAAAAVGGPTDQ
IRGTFRRAMGGGFGFVRPSSGGGNADADVPEDVFVPTGMTAGALEGDLVAVTIEPSRRGGIEGKVVEVLQRARRQ
FTGTFFSSPQPDQPGSDTIEGPVVYLDGVHYEAPVSVGDVRGLPLQDGDKIFVEIVDFPDEESGGGEAVILERLG
SSKNPAIDTLTIMRQYALPDEFSEDVLDEAREQADAFDDDVVPTDRKDLTDMLTITIDPFDARDFDDAISLQRED
GRWRLWVHIADVSHFVPPGGKLDVEARRRGTSVYLPDRVIPMIPEIISNHLASLQPERMRLVKTVEIEMLDDLTI
THSEVHNAAIRSDKRFNYEQIDQFIASPAAFQKDWGDSICELLTHMHTLAMQIRKRRFKDGALSMDMPDIKLELD
RTGKVKGAYQTENTESHQIIEEFMLLGNEAVATWLDDQELNFLHRIHAPPERRKLRQLTSFVKDLGLGFDNVESR
FEIQAVLDKVAGTTLENAVNFAVLKSMSKAVYGPHREGHYALDKEHYCHFTSPIRRYPDLSVHRLVQRLIEQKST
PDESFAELVKLGHECSDAERNAAQAERELIQLKLLHFLKKKQGETLEAVISRVFADGIHARCLKLPVDGFIPVTE
LPSDQYRFERRGQSLTGFKSGNRFRLGDHLTVRIGKVDLQDRQLYLEVVKNHSAAKSDPRGPSGSKSKKSPLRHQ
KKSDRREKKKRRRR

ORTHOMCL122
>gi171910496refZP_02925966.1_pro GTP-binding protein TypA [Verrucomicrobium spinosum DSM 4136] -KR.{10}KKKL
MSPANIRNLAIIAHVDHGKTTLVDTLLRASGNFRENQQIAERAMDSMDLEREKGITIKAKNTSVHWNDHIINIVD
TPGHADFGGEVERVMKMVDGVMLVVDAYEGPQAQTRFVLRKALQQGLTPIVLINKMDRPHIQPEKVHDNVLELFL
ELEASEEQFNAVFLYGSARAGWVSDSPEGEHHAMEFLLEKIVKHISPPKAEPEGSFEMLVSNIDWDNFVGRVAIG
KVTRGSVKMGDRVYLLGKTPEESAKAIKVTKVFQYTGLGVSENAEGTAGDIVGISGFEDVDIGQTVAGSADAEGL
PFVAIDPPTIVMQFAVNDGPLAGREGEHVTSRKIRERLFKEAKMNVSISIEDTDLAGVFNVSARGAMQIAVLVEQ
MRREGFEVVISRPMVITKRVDGVLCEPFETLYVEVPEDYVGGVMKSLAERKGRIEDMKTHAHGSTLVATVPTRGL
IGFEFELMNLSSGHGIHSHLFKEYAPHAGPMQTRSTGTLISTESGEATTYALDTIQVRGKLFVAPGDQIYDGMII
GENPRMDDLPVNPTRSKQLTNFRAAGNDKSAALPPPIRFSLERAIEYVAPDELVEATPKSIRLRKRTLDSTVRAR
EKKKLEAEMEAV
>Blastopirellula_marinaGL01984_int gi87309243refZP_01091379.1 translation initiation factor [Blastopirellula marina DSM 3645] -[KR]{4}.{20,24}K{1,4}.K
MPIRIYALAKELDIDSKELVEICNRAGVTGKGSALASLTDDELDKVKSYISGGGKKQDRKAKASGAAKEAVIDQP
IREERPVQAGRPKEIRTIRTGNAPLAVHGRPARPSDVSVEEQPEEQEAAAEPEVEATPPVEAPVAETVKPPAPPE
PEIKETVEVEAEKPVEPPAAPEEAAAEINAIRRTDYIAPGGGGRGKVRVLGTTRAPDGPGDASKREAANKPKRVA
TTVKMAKMPTPAAPTQPAKKSGEPAPQKPIMQLPKAAIQGAKAGQKAPLEEFTKQHEKKRKDKERRPTDAPVADT
DEAAGAGDRRKKSKGLAAMAGGREARAAGRRTKKPGEDDSGDRMRRRSRSRTQRKGANTAAPRKSNVELELPCSI
RDFSEATGIATIKILRTLMTLGTMANINTIIDTETGEYLAAEHGVEIDLSEAQSVEDKVITKIEEMEDAPDSLHE

RPPVVTFLGHVDHGKTSLMDRIIGHNVASGEAGGITQHIRAYIVDKDDKKIAFVDTPGHEAFTEMRARGANVTDI
AVLIIAADDGIMPQTEEAISHAKAAEVPIIVALNKIDLPGANPDKAMQQLSQHGLLPTEWGGDVEVVKTSAVSGE
GIDALLETILLTAELHEYKANPDRPAVGVCLEAEQESDRGVLAKVIVKNGTLKLGDVIVCGPAHGRVKAMYDTQN
GRKRLTEAPTSTPVNITGLDQAPGPGDKFYVLDDIAQAREIAELRSSRSRAESLGVHKVKISFDEFQERLESGTL
GGDKSVTTLNVIIRADTRGSIEALQKEMEKLDHPEVKVRVLQAAVGAVSVADVTLASASEAVIIAFNVIPDEAAR
SLADDKGIEIRRYNIIYKVTEELKLLLEGQLRPEERINELGRALVQRVFVVSRLGSIAGCRVLGGIIERGCRIRV
NRDGRTIGDYPLESLRREKDDTKEVREGYECGIRLQGFNDIKDGDILEAYKIEEFARTLD

ORTHOMCL130
>Escherichia_coli_O157GL00241_pro gi15829499refNP_308272.1 H
repeat-containing protein [Escherichia coli O157H7 tr. Sakai]
-RKRKK||[KR]{4}.{20,24}K{1,4}.K
MSIQSLLDYISVIPDIRQQGKVKHKLSDILFLTVCAVIAGADEWQEIEDFGHERLEWLKKYGDFDNGIPVDDTIA
RVVSNIDSLAFEKIFIEWMQECHEITDGEIIAIDGKTIRGSFDKGKRKGAIHMVSAFSNENGVVLGQVKTEAKSN
EITAIPELLNLLDLKKNLITIDAMGCQKDIASKIKDKKADYLLAVKGNQGKLHHAFEEKFPVNVFSNYKGDSFST
QEISHGRKETRLHIVSNVTPEFCDFEFEWKGLKKLCVALSFRQKKEDKSAEGVSIRYYISSKDMDAKEFAHAIRA
HWLIEHSLHWVLDVKMNEDASRIRRGNAAEIISGIKKMALNLLRDCKDIKGGVKRKRKKVALNTCYIEEVLASCS
ELGFRTDKMKNLTQI
>Singulisphaera_acidiphilaGL08226_int Isosphaera pallidaGL002127
locus=Scaffold121525116378 -PRRRK
MANDLSTRIEDHFASLTDPRRRKVVYPLISILTIALCAVIAGADDFATIAAWGRQKRAWLAKILDPTNGIPPHDR
FNAIFRAIKPAEFERCPLSWITSLHEVTAGQVVAIDGKTLRQRFDKANAKSAIPMVSAWATTNHISLGQIVVDAK
SNEITAIPKLLELLDVSGCLVTIDAMGCQTEIAEKIVQGGADYVLAVKGNQPTLFEGIMNFFVGHMDDDFARVKV
SRHETKDKGHGRIEHRTYYVCDVPEELPGQARWKGLKQIGVAISDTMRGDKPCDDVRYNILCKKLSAKSFGAAVR
SYWKIENSLCWQLDMSFGEDRSRIRNGQADANFAIVRTMALSLLKHERSHKSGVKSKRLTAGWNDDYLEQVLFGS

ORTHOMCL147
>gi171912313refZP_02927783.1_pro glycosyl transferase, family 2
[Verrucomicrobium spinosum DSM 4136] -KRPRP
MPESPVVLHAIEFPVERKLHSRKLTIHGWCFDLTGEFLKGVRATVGEQIFTARRKRPRPYLGYRYPDHPEAAVSG
FQIDVEVPRGRSIIQVECKRSDGRWVLLEELPVAAPWIAWPWKQKVSGQDYETWVHHYDTIHADDLVKLRQTGAA
LTDGPLISVLLPVYNTPLRWLKRVIETVLGQAYPKWELCIADDASPDPQVRKTIESYAAGDPRIKVVFRPSNGHI
VAATNSALELATGSFVALLDHDDELRPHALLEMAREIMGNPEAALIYSDEDHLDEAGVRYAPYFKPDFNYDLLLS
QNCVCHLGVYRADLVRQLGGFRAGTEGAQDWDLALRVFEAVGRDRIHHIPKILYHWRSIEGSTARGVSEKSYASS
AGRKVVEDHLSRTNQAVEGVEEVKQGHLRVRWSLPNPPPVAIIIPTRNFRHLLEVAVESVLARTDYPNFRLVIVD
NDSNEESTLDYLASLQAQGKADVLRIPGPFNYSLLNNRAVAACTEPVVCLLNNDIEIHERDWLREMVSQAVRPGV
GAVGTKLYYPDGRIQHAGVILGMGGAAGHFLKGCDSSNESHGGRLHVCQNFSAVTAACLVVERRKYLEVGGLDEG
DFKVAFNDIDFCLKLDDAGYRNVYTPFAEMSHHESASRGAEERTDAGKERATRETMLLRQKWQPYCEADPAFNVN
LSMVHEDGSLGFPPRIGEWQTRREKRRSESSALLPPRGGGLG
>Gemmata_obscuriglobusGL06262_int gi168704132refZP_02736409.1 hypothetical
glycosyltransferase [Gemmata obscuriglobus UQM 2246] -RKRRR
MSERTSRKRRRGGNPYAGLIHLLNQRYHAEWQRAELLQNELTGRENSKVARAAEFVRRFVRRFVPHPAVVPKVVT
ELAVPYTGGARAVPDATVSIIIPFRDRPELLRNCLRSLRRSTYKKTEVVLVDNGSEDPRTARLLAGISAQRNIKL
VRCDEPFNFSRLCNLGVRKATGDHLVFLNNDTEVITRRWLERMLVLAADPAVGAVGATLLYPDRTIQHAGLFPRS

DGAWVHPYRGEPAEAVGENGELRVMRIVPAVTAACLLVRRDVFESANGFDEDLQDSLNDADLCRRLGAAGRVTVI
TPHAKLFHYEGLSRAFNVDPLMA

ORTHOMCL167
>Thermoproteus_neutrophilusGL01207_pro gi171185692refYP_001794611.1 ABC transporter [Thermoproteus neutrophilus V24Sta] -[KR]{4}.{20,24}K{1,4}.K
MSAVLEVRGVYKRFGGVVALEDVSLAVAPGEVVAVVGPNGSGKTTLVNVISGYVAPDRGSVLLEGRDITRWSAER
RVRAGVVRSFQFPLLFPNLTVEENLRVAASGLKPYTLGEVDRRVVEEALHLFDLEAVRRRRVTELSEGHRKLLDV
ALAFVFKPKVVLLDEPTSSVSSQEKFAVMEKIVEVVKKSGASAVVVEHDLEVVKKFTDRVVQMMAGRVVKVAKTA
EWSHD
>Isosphaera_pallidaGL01409_int gi320102964refYP_004178555.1 ABC transporter-like protein [Isosphaera pallida ATCC 43644] -PRRRK
MSEPAVSVPAESDVSSTSTATTPSKRSCRWGWNLWRRLGVEASRRKRQRAQSDRRVRTPVYLQMEASECGAAALG
IILLHFGRYVPLEQLRIDCGVSRDGTKARLMVEAARKYGLEVKPRHYEASQVRRLAPPFVVFWQNNHFLVVEGFR
RGRVYLNDPASGHRVIDDEEFDRSFTGPTFRFERGTLKPGGRRPSLAEELIRWSRGAGGLWSLVFALGLMGVVPS
ILSAVFGKIFVDAILIAGQWDWFRPLLIVMLFTMIARVVLLWLQQNRLLQLELGLTTRQTSRVLWRVLHLPMSFF
HQRFAGDVASRITANPRIARVVAGEFATLAISLLMVVFFGVVMISIDPQVALTGIALGSLNLLGVQLVARWRSEE
KQKISQLQGKLTASLTFAVQTIETIKASASEADMMVRWTGFQTRLINARQRLGMADAALLALPATLGVATTAAVL
GLGGAHVVEGTLSIGGLLAMQTLLASFLQPFNDLVRIGTTTQELEADLNRLNDVIHHDPDPTTLPPSHSVGSAAR
ASPPLPPSWTDLTASPSPKAPPAGGESATVVATCAAAWHPVNIETPVVRPARRPPPRLSGLVQVREVTFGYQRYG
PPLLEGFNLTIQPGGRIALVGGSGSGKSTIGKLIVGLLQPWSGEILFDQRPRAALPRATMINSLGIVDEFTFLFA
GTIRENLTLWDETIPEEMIQRAAIDARIHGDLIKRPGGYASPIREAALNFSGGQRQRLEIARALTRDPSLLILDE
ATSALDPKTEEEVDDAIRQRGCACLIIAHRLSTIRDADEIVVLRRGKVVERGTHEELLDLRGEYWNLIQQDESSI
GRDPQGQATQGQSLPRRRKTGRTHHAQVTSSRRAAIIHPSPEDALASGWTGSAKESPERDDSLTPLNLERELDRS
DRTSRLVVSGRSPVPLDDPNRVWQVIGGKVDVFLLQSEANQAAKTRRHLCRILEGGAIFGVGATRGPAGASFLAV
GVGEARLRSVHRGDLIRLGLEDDLRWQLAQWIDGWIEAMSRGLAGEPPRFRVEDLKDDLVLAEGDLARPRSGVVW
VWPRQGSLRFLGEVTIPRNEDEARFPLAPGTWIQAETASILRCRSTERLLEDQDPWIGLKLFHEVVLRYAAQIGR
GVAMRRNRIARASSLRDEAALGNALNRLIAPLQERNNHVPIDVGGGQPLLAAYRAVASVFGIEVKPPRSLREGQG
VSDPLTELGHASGLHARRVRLRSDWWRSAAENGPLLAFLEPLRRPVALLPNRSGDGYDLFDPSHPGRIPYPPPSS
ARTHDQPAAVTHRNLATNGSTTTNQHAGFHGESQPRIQFHPRTWWRRKRPARRSAGRCREPSRESNAPISASAQS
PGQPVALSPYAYRFYRGLPGHTLTWRDLLTFSWPVIRGQLRWILVLGVVVGLLLLIPPIALGLIIDQVVPAAELD
RLALLCGAITVIALMIGAFRFFQGRAVLRIEGLLATHLLPAIWDRVLRLPTRFFAEQRAGDLAHRLLGFETVLGL
LSGATVNTVLSFLSSSFALILLFWYQPILALVALGLVVAMTTITFFLIGRYVQIQRRIRALEGEVSSLLLELLAG
IARIRVAAAERRAFARWSEVFVKQVEQTRRGRVIANRLALFYAAFPLATMAILYAATVSLVASGLEVGHFLAFNF
AFFGFVGAALSLGNLLASMVSVIPIYDRIRPVLETPVEQEHPGAEVGCLGGAVSLSRVSYRYHDDGPLVLNQVDF
QVLPGEFVALVGPSGSGKSTLFRLLLGFDRPTEGVVAYDGKDLATLDLYDVRRQLGVVLQNTQLMPGDLYTNIVG
FSSSLTMEDAWEAARLAGIAEDIAAMPMQMHTVIGEGAATLSGGQRQRLFIARALVKKPALLLFDEATSALDNPT
QRKVSDHIASLKVTRVVIAHRLSTIMGADRVYVLKEGRIVQKGRYEELIREPGVFREMALRQQLLREEG

ORTHOMCL175
>Phycisphaera_mikurensisGL02456_pro gi383767531refYP_005446513.1 rne gene product [Phycisphaera mikurensis NBRC 102666] -RKRRR
MLIDYVPGEECRIAVVEDGKLDEYYHERASSESHVSNIYQGVVTNVEPSISAAFVDFGLERNGFLHVTDVHPKYF
PGEDREAFEKVGHKTRHGDRPGIERCFKKGDQVTVQVLKEGIGNKGPTVTSYLSIPGRFLVMMPDMQQLGVSRKV

EDDEQRREMKQLLKSLDPPKEFGFIIRTAGLGQSKVDLKKDLSYLVRLWKDIEERVAGDGGGKGKRKKGRRRARV
TRELYTESDLVIRTIRDVFTPDIDRVVINHPEAAKRAHDFLAVSNPRAKSKVVFYDDPVPLFHRFGLEQQIDNIG
SRTVELPSGGALVIDPTEALVAVDVNSGRSRSAKTSESNAFETNKEAVDEIARQLRLRDLGGLVVLDLIDMFQQR
HRRAIESRLKKNFLKDKARTRVGGISQFGMLELTRQRMRSSLTEAVHQECAHCGGRGHTKSVESVVLDVMRQLAM
VMQQPQVARLELTISPDVAFHLLNRKRAEMVALEAKWQKAVTVRVGGGSVDYVQVSPLNEQGATLAPTAASAIPK
ETETTFRDVDALDDADLEAWMAPVAVQVEDPDVLGGHARAGGIDDAPAAAQEEAAGSGARAAADDPTEEKSQRG
VGDDEEAERPKRRRRRRGRNRGDEDQEQAPGEASAPERDHARRRSAGGSSSESRHEEARRPEPEPQRAASKPPTG
PHRDPLLDFEFDPQSHPLDRARAYAEHRLRTGAGEGPLPEPGSAAKRLEEATGTVSVPVARPDGDRSGPGSEASG
EAAETDGPAGDAGAGAASEGGLTATEGEGTGKRKRKRRRRRRGRGDGGDTGDSASGEPGQAADDRPRDEHGDIDG
NVRVQDPAAGPDPAEEDDSRGNRHEAPPREAPEESAERAEPRAAEAAEAAEAAEAAEADPAVQPEAETQAETEAK
SKATTKKKSKSQRTRKPKPAPAELEAEGSEEEPPKPARKPRTRKSPAKKSPAKEKAAKEKASGAGASEPAETAEA
TKEAEAAPPKKKRRSRKKAAATEGGGGSDADGEDA
>Zavarzinella_formosaGL06870_int Gemmata obscuriglobusGL008929
locus=Scaffold7319910322582 -[KR]{4}.{20,24}K{1,4}.K
MKKEMLINVMQSEECRIAIIEDGVLQELYVERASNESYVNNIYKGKIVNIEPSIQAAFVDFGIGRNGFLHVSDVA
SVYFKDQPSGDGRREDRDDRPRRDDRPRRDDRRDERPRREEPQPHSHAERELLPPRQFEPVEIPHIPAAPVAPPA
PPVYVAPPAPVSPPEYSDDIPMAEVYEEDDFGAGLDIGPDVSSPTAFPAPAPAPVHVPEPEPVQEPVHEERNPVA
DPTEPAEPVSESAESGEADADSEAKPKKKKTTTTRGKKKAAEPEPVVEEAKPKRKRTTRKKTEGEEGGEDDGAKP
MPSAERRSTGGDDEFYFDPMAPNDRFGGGEEPKEEAPEGEIGFGESEEAVGEVVEGDQLFEEGEPGDIEPQPIIP
QGDEEFVDEFPVQDRPERGRGGRDDRGGGRGRDDRGGRGGPGGSRGADRGRDGGMKPPPIEQIFKRGQEVIVQVI
KEGMGTKGPTLSTHISIAGRYLVLAPWLNRVAVSRKIDDDSTRGRLKEIMRELNAPSGIGFIIRTAAVDRNVQEL
KSDLAYLVRLWEVFTSRTTKRQSPIEVYRESDMITRTIRDMFTSDIETIYIDDPEAFAQAQEFMKIVMPRYADRL
KLHESTEPLFHSFKLEEEVHKLQEKKVPLPGGGSLVIEQTEALVAIDVNSGAYRAENNAEETAYQTNLQAAKEIA
RQLRLRNLGGVIINDFIDMRQESHRRAVEETLRKGLRRDRSRTKILKTSAFGVIEMTRQRVQTSLKKNAFAECSH
CRATGLVKTPETMSIDVIRMIQLAAARKTAKVVDLHVHADVAHYLLNKKRRDILKWEDHGGMTVSVTGRIGVSPE
FLEVRGFDNNGHEVPMQLAFSAPAAIRPPERREERRDDDRDDRGDRGPRGGGGDXXXXGGGGGRR

ORTHOMCL180
>gi171913997refZP_02929467.1_pro two component, igma54 specific, transcriptional regulator, Fi family protein [Verrucomicrobium spinosum DSM 4136] -RKRKK
MDILVIDDEKLIREATMQIVEDAGHYAESAQDSATALEAIKESTFDLVLLDVNLGREYGLDILDEILKRNPQLPV
VVFTAAANISLAVEAMRRGALDFLEKPFTGTQLGLVITRAQKHRKLAERVVELQTQVASHSPEPVFDSSNAAVKD
TLDVLFRAAPTPASILILGESGTGKSVVARAVHQRSDRANKPFITVNCPSLSKELLESELFGHTRGSFTGAVKDQ
WGKVKAAEGGTLFLDEIGELPLEIQPKLLRLLQDREYERLGENTIRQANVRIIAATNRDLAKAVGDGTFREDLYY
RLNVITVTMPPLRQRQQDVMRFSEEYLKFFAKQCARPLPAFSEEARRRVMAYPWPGNLRELRNVIERVVILAASR
EIKATDLPPSLNGSAAAESSADGPNKGDWVSLEDLEAAHIKAILSRTHSLAEAASVLGIDQATLYRKRKKLGLDS
E
>Zavarzinella_formosaGL03502_int Gemmata obscuriglobusGL005465
locus=Scaffold3160746162074- -RKRKK
MNILLVDDEASLRRTLRTTLEAQGHAVREAATGAAALRQLDEQQADLVFLDLKLGRESGLDLLPKLLEASPGLGI
VMMTAHASIGTAVEAMRRGATDYLPKPFTPDELRVAIDRWHTVRSLRGRVARLEEQVRQTVPDVELTTAEPAMET
VLKTAFTVADSEASVLLRGENGTGKGVLAREIHARSRRKDHPFVVVHCPSLSAELLESELFGHARGAFTGAVEAT
EGKVFAANGGTLFLDEIGDLPLGLQPKLLRFIQDREYERVGETKTRTADVRLIAATNRDLEAEVKAGKFREDLLY

RLNVIEITMPPLRQRRHDILPLAGHLLKFFARQSGKSMTGFTAEAREVLMKHAWPGNLRELRNAIERGVILSPGG
EVGVDQLPPGLGSRKARLEVGGLISLEELEAEHIRRVLGATASLEEAAAVLGIDASTLYRKRKKLEP

---

euk+pro+int
>Phycisphaera_mikurensisGL03124_pro gi383768199refYP_005447182.1 unnamed protein product [Phycisphaera mikurensis NBRC 102666] -KRPRP
MLIEPYVTPAGALAVREAEGGETAPSVAAAKRLLTAADAEAGGGSGAALLELGAGWADAELGPRLGWLRAFARAA
LTAWCRAAAQEAGAVETPSEAALAERVTHAPPLAGGEYLRPETLAAWWEQTGVALHAAADAAGLPIGEYLASRHA
SWRAVGRVTFHLAENQRDEARPFAFLATYADGPAPDGTPRHRPLGKAIETSAGSGDRATLLGLLTPIYRAAERAD
WLREAVVSGAVYRPLAWTTGEAYAFLRSVDTLQAAGLAVRTPDWWSAKKRPRPTVSVQVGGTKGGAVNTASLLSF
SVSVTLEGETLSPEEMDELLSGGGGDAAGLVRLRGRWVELDRDRLGAALDHWKEVEAAHAEGVGFSEAMRWMSGA
QAVAEEQEPSAGADPEDPAAWVGIEPGPWLEETLATLRDPACLAPAEMPGLKARLRPYQATGVGWLGFMSRLKLG
ACLADDMGLGKTLQVLALLLRMKRADAEAGPDANLPPSPSPPPSLLVVPASLLANWAAERDRFAPSLQSVTLHPA
ESAVDLKDAGAVKRAVAGVDLAITTYGMLTRLDVLRELPWRLVVLDEAQAIKNSGTRQTHAVKKLTAESRVVLTG
TPVENRLSDLWSLFDFLNPGLLGSAAAFKRLVKQMGGDDDRPADYAPLRRLVGPYILRRLKTDKSVIADLPEKTE
VQAWCGLSKPQAKLYQASVDEMARELREGERAGIHRRGLVLGFLTRFKQVCNHPAQFHGTGAYAAEESGKFLRLR
SLCEEIAARQERVLVFTQFREVTAPLAEQLGEVFGRSGLVLHGGTPVKKRQQMVQRFQAADGPPFLVLSLKAGGT
GLNLTAASHVVHFDRWWNPAVENQATDRAFRIGQKRNVLVHKFVCRGTIEEKIDAMIQEKTAVADAVLGGEAEKR
LTEMDDVELLDFVKLDVARAGEL
>Rhodopirellula_balticaGL05170_int gi32475836refNP_868830.1 SWF/SNF family helicase [Rhodopirellula baltica SH 1] -TKRS...M
MSFHDLKTKFESLDGLEQLSCQLLAIAFDTVDLSDLTKMVRHALPCLTKVGKPTPPKVTQAIIKPALKHLQNIGC
IQSQSNLRFHIQPSVADAILRSSIHEGTFSDLVFIVNNLATQNRFPWRQIHQSEQQKRQRLAFYQGDVESYFNLA
PEEAIHTRKLGLLTPFDETVYEGLPIELQDAYWPVFGPSLLHTANGSTGMMDHVEAWLKRSSPDDEVAISVGTDL
LFAAGRCSTLQPLAELLKNQRPEILGLVCLLQGDRDQATQHFATAMPATSLGKSSSLTACQTFAGLYYPLLLLRQ
SDPQATATAQKIIQRRIKKLDQDLLLGCDAVLSGCDFLEDPGLAQHIQASWTDDVSRPLATLLSGYVREICSSSE
TASRELNNLAQIGKLYLASGLDWLAAEAMTLVNEHKPNQSTAKTIKKIYNTTGTSPMAGQFERSPIWKKQLGALQ
AFAASFQPASSPAQQAGQATPQESDRLAWLVNIAASGRSLGCRPVHQSYSKKGWSKGRPIALERIYDPSRLAEFD
FLKPEDQKICGSLRCETSRDHYGYTEYHYYFETALLVDALIGHPCLFMDDDARSPIEIEKNHARLVVERSPEGGA
TISLSPRPTSRESHLLVHRESTSKFSIVQFSDAQLKLASMLADGLTVPDSAVDQLLDAVRPLAPIAPLHSEIEVT
ADGAEQVVADNTIHAHLTPYDHGLQIAFFTRPFGDSGGFFVPGQGSRTVVTKINGQVRSTRRDLEAESESLRAVQ
NDCDFLGQQADAEVIDLPTAAEALEALLELNSLVEESRLTLHWPKGQAFQIAGSADESDWKIKINGGQDWFAASG
KLHVDSNLAIEMMDLLEMASASTSRFVQLNDGRFVALTEHFRKRLETMAAYTDRRQDQVRFPTIRATAIAELTGN
SDLTADQQWKSQIKRIESADRVRPKLPKTLNADLRDYQVDGFKWMSRLAHLGAGACLADDMGLGKTLQCLAVLLN
RGKSGPALVVAPTSVAANWVSEIARFAPSLRPILFSEADRETVIESLGKRDLLICSYGLLANEAEKLQSRRWQTL
VLDEAQAIKNADTKRSEAAMGLEADFRVVLTGTPMENHLGELWNLFQFINPGLLGSSESFQERFAIPIERDHRRD
VQRQLKQLIAPFILRRTKSQVLDELPPRTEITVPIELGEDEAAMYEAMRRKALQNLEDSDDDRPVHIKILAELMR
LRRFCCHPDLVDPDAGLKAAKLERFTDTVTDLIEGGHKVLVFSQFVGHLHLLRDRLDERKISYQYLDGSTPAKKR
KTSVDAFQDGEGDVFLISLKAGGVGLNLTAADYVIHMDPWWNPAVEDQASDRAHRMGQQRPVTVYRFITTGTIEE
RILQLHESKRDLADSLLEGTESSAKLSAEELMKLIL
>Eremothecium_cymbalariaeGL01096_euk gi363749913refXP_003645174.1

hypothetical protein Ecym_2645 [Eremothecium cymbalariae DBVPG#]
-K[RK]{3,5}.{11,18}[RK]K.{2,3}K
MDPDTPESTYENSDKAQVQVPESSPIESKQDSSPLKAYTPPAKRDKPFVALLHDKFTYKPNISNGHVEVSGNVTG
TSTGVDTHLNEIRQHYPDFSDTLIRAVFKSNSFSIPLTIDRLNELREKKMAKSWAMPSKKIGSSTSRLPSLKRPS
SSGSMTGGFDAMSSASAMKQHVIVASPERSSKITLEKQKKSIFDRYSHVMHRGKLDPNEPLQIKTIPWQADAAAV
KKRKLVRADNIVTNPKKVKKDKISLSDEDEDEISADEGSADEFSGDDYEERTPEVNIDDQILIFLNEADSSDIAD
LADTSIEKAQIVVTKRPFKSLNSFSMMDFAKNDKAAQVDNKKTTRRRGPMKKDGAKMLEKITQSMKGYNAIESLI
KKCSSYGVLIANQIRRWGVKVEENGSGELTFVDLKISGEKSSNSDGENGYDKLSIQGRREPTEVSSGKSKRKRQQ
EDGISIGSIKERQQEEQEEDEDEEEEEEYEDDEEFEDVYEDSGDEEFVAIQRKNNSGRNRGRPEKKDVSFFRRKP
KLLGSDVILKDYQQTGINWLNLLYQNNLSCILADEMGLGKTCQVISFLAYLKELNERGPHLIVVPSSTLENWLRE
FNKFCPQMKIEPYYGSQQERAELREILEENDGQYDVIVTTYNLASGNKADVSFLKNRNFNVVVYDEGHMLKNSTS
ERFTKLMKIRANFRLLLTGTPLQNNLRELMSLLEFIMPSLFVSKKEELAAVFKQRAKTSDSNKDYNPLLAQEAID
RAKTMMKPFILRRRKDQVLKHLPAKINRINYCDMNAVQKFIYNKEIRLVMEHKRMLKEGLLPADAKERAVVTHST
SKNLIMALRKASLHPLLFRHIYNDKMIHDMSDRILDEPEYAETGNREYIREDMSYMSDFELHKLCCKFPNTLADY
QLKNNEWMSSGKVETLSVLLRDIIDRKREKVLVFSLFTQVLDILELVLSTLNIKFLRLDGSTQVNDRQAIIDKFH
EDPDISVFILSTKAGGFGINLVCANNVIIFDQSFNPHDDSQAADRAHRVGQTKDVVITTLIARNSIEEKILQLAK
NKLALDTSISAEDSKNKDDDLETRVSDLLEDIIYDEHTK
>Eremothecium_cymbalariaeGL01582_euk gi363750886refXP_003645660.1
hypothetical protein Ecym_3355 [Eremothecium cymbalariae DBVPG#]
-K[RK]{3,5}.{11,18}[RK]K.{2,3}K |[KR]{4}.{20,24}K{1,4}.K
MSLDALLNKDELTSMADGGGMDGVCSSLVGSRERFMSSMNERFNGLCHKDRREQQYQDWKYLSYQEFELVNEWSA
QSRDLTINQCDQLISNIKSAQDEWMDYEVFLSGRSTVLGELEEKRQKEMEAQEERQRDRDHLDRQQDKQERQQQQ
QQQQQHPQSTKIRQERQRSAATANKRSAKRSGGSGNSVAFSGSKAKAETSVANRNHQQVGHGGSAATELNGAVKE
LYQGKSHNRGKGDEEPRTIESLKQGDEDREDKDEDEDEDEDEDEEDDEEEEEDDVDELDELEEIQLMDDDNDRDF
SPESGRSKSSVKFNSKIDVNSDVNLIQKELIKMAQKHRNAKAKKRKFTSCIVQKYDADHSKLEVKVTLKQLHIKR
LKRLINEAKRKRAAEEAVVANEQQAGSSKKRKTGQKSKSNTSGNAASSNETNSAESPAIGANTKVNGDANSSDSQ
VPVVPDINPTTGLPTYGMKMTAKEARAIQRHYDTTYITVWKDMARKDSAKLSRLVQQIQSIRSANFKKTSSLVAR
EARKWQSRNFRQVKDFQTRARRGVREMSSFWKKNEREERELKKKAEREAIEQAKKEEEERESKRQARKLNFLLTQ
TELYSHFIGSKIKTNELEGSMADPSLSMFTAGIDLSNTDPTKTEVHQIDFYNEDDEELRRKAAQNASNALKETQA
KAKAFSDPSDEAEELNFQNPTSLGEITIDQPKMLGCTLKEYQLKGLNWLANLYDQGINGILADEMGLGKTVQSIS
VLAHLAERYNIWGPFIVVTPASTLHNWVSEIQKFVPDFKILPYWGNGNDRKVLRKFWDRKHLRYDRDAPFHVMIT
SYQMVVSDAAYLQKMKWQYMILDEAQAIKSSQSSRWKNLLSFHCRNRLLLTGTPIQNSMQELWALLHFIMPSLFD
SHDEFNDWFSKDIESHAQSNTQLNQQQLRRLHMILKPFMLRRIKKNVQSELGDKIEIDVMCDLTHRQAKLYQVLK
SQVSSNYDAIENAASSISGDDSGNYPATSDSKIMNTVMEFRKVCNHPDLFERADVSSPFSFTNFGQTGSIIREGD
VVDVQYSSKNPVTFNLPRLIYDDLILPNYKHDIDVRAKILYHSMNIFHPTNSPGLCSTLSKVTGYDPDSFMHLSN
LEIVKRAIHLSSNCANKKDIIPIVYDKEDFNMNLFEKSLLVTNEVDYLRNLSCTTRNGVLESLLNIKDKVYDEQY
MNVLRPAYRPSVAAPPINIHVMGSRNFMIRRDHELFEPEICQSLSIVPPITQNSLVQRNKEMLFTELPKSELYPS
PLNKSFSSYISMPSMDRFITESAKLKKLDELLVQLKEGDHRVLIYFQMTRMMDLMEEYLTYRQYNHIRLDGSSKL
EDRRDLVHDWQTKSDIFIFLLSTRAGGLGINLTSADTVIFYDSDWNPTIDSQAMDRAHRLGQTKQVTVYRLLIRG
TIEERMRDRAKQKEHVQQVVMEGKTKENNVQTVLASGSSIDPLPTPPAIEVVSDTTV
>Penicillium_chrysogenum_WisconsinGL00987_euk gi255931991refXP_002557552.1
Pc12g07170 [Penicillium chrysogenum Wisconsin 54]
-K[RK]{3,5}.{11,18}[RK]K.{2,3}K |RKRRR RKRRR
MTGPPPYNAQSPTQQQRYPAYTSPNKNHPYYPSNEQPQPPQYHQHPPQTPPAFGPSSVARSPHFSHASPMPSTLP

PPLNGSAPPHPSHSEASQYQGHSAPGSQLPLPRPYSSSVITGNGASPYGSATPHGHPPSRPEGHSQSPTRESESP
YCMRGNGAGYGPPMMREPRPVSPQEAKPARAADPMSFASILSGPSDEQPVRKPSPLPAPISTHTPTPHAPPVFTH
RHLDHNSTPIAMHHKTEHRFDFDKRIDGPRAPQATNGFVKPAAEYPAPVARAPLRKPFPPGVDLEQVNRAAAEID
RAEKSDIEDPAFDAEQERYREKGYKRSVESSRAEEARRKRRRNEFLIDLGRSFEKQAILGTERFRVLNEGAVMAE
VQQKEIQDEKERKKDMQRKRRRENTVRQEMQKKLEAERKASKAQEPGEKAKFLREAERAQKKIRTTKRALEGGDG
QDELGEVTPLAPNLEGGTTSQFHIGRSSPSRRRSGRAGPVTRPKKSKEQKQAEKDLAEAAWNAGLDDDLYLTPST
RKDARRSKEGTPMSQLHYDSKGYNQIYEQIWRDIARKDIPKVYRIKTTSLSTRQENSRKTAQLASKQSRKWQERT
NKSTKDTQARAKRTMREMMSFWKRNEREERDLRRVAEKQELESAKKAEADREANRQKRKLNFLISQTELYSHFIG
RKIKTDEAQGDGAVAATGETVQPGKPNAHTVSLPDSVANPNAKTTAFEDLDFDAEDETALQQAAMANAQNAVQEA
QDRARAFNQGEGDNMAAFDDGEMNFQNPTSLGDIEISQPTMLNAQLKEYQLKGLNWLVNLYEQGINGILADEMGL
GKTIQSISVMAYLAEVHNIWGPFLVIAPASTLHNWQQEITKFVPNIKVLPYWGNAKDRKILRKFWDRKHITYNRD
SEFHVLVTSYQLVVLDAQYFQKVKWQYMILDEAQAIKSSSSSRWKNLLGFSCRNRLLLTGTPIQNNMQELWALLH
FIMPTLFDSHDEFSEWFSKDIESHAQSNTKLNEDQLKRLHMILKPFMLRRVKKNVQQELGDKVEKDIFCDLTYRQ
RALYSNLRNRVSIIDLIEKAATGDDTDSSTLMNLVMQFRKVCNHPDLFERAETKSPFSTAYFAETASFVREGNFV
DVRYSTRNLIEYELPRLLCSSTGRLDLPGSDNPRVGFQTKYLSQLMNVWTPENIQKSARENGAFSFLRFVDTSAG
EASEMARLGVYERAERRRSKPNRLSALNVLYDESDDSGNSVLPHSMLNIVDRNDRQAVREIAAEGRMKDLMNVSR
CSFEDQGLHVIEPCAAPKASAPPITMSSSGQQAHRESHHALFNPSVRQALSGYCSRQVEEQILIKKLDPAPYSHA
PMLPPPMSLKGRYNHIEVPSMRRFVTDSGKLAKLDQLLRELKPGGHRVLLYFQMTRMIDLMEEYLTYRNYKYCRL
DGSTKLEDRRDTVSDFQSNPEIFVFLLSTRAGGLGINLTAADTVIFYDSDWNPTIDSQAMDRAHRLGQTRQVTVY
RLITRGTIEERIRKRALQKEEVQRVVITGGAAGGVDFNTRARENRTKDIALWLADDDEAELIEQKEKEAIERGET
LGTKGGKKAAQKRKRDLTLDDMYHEGEGNFDDASAKPSGAATPAEEPVETPSSTPAKRGRGGRGGGKGTSKRAKT
TKERLRLIDGDGGLD

>Gibberella_zeaeGL02076_euk gi46110288refXP_382202.1 hypothetical protein FG02026.1 [Gibberella zeae] -RKRRR

MDQNGYNSSALQRPPRRGDEGCEEDRDSRPHHHHRHHHHHHHHRRDGDLPAGAVAGEAATASSNAGGANAHQHST
FSLRSPKPEYRPPPFSSPNGHNHSHHNTSTSSANHSLQSPPRPALPNPYMSSSTGAPGGPVAPALPPPVGINSSS
SPGSSAAGLHQRHHQPGAPAHQHRAAPPPVSPLHPPVAYYPPGTNTDIYIPPPEPKPASRGFYDPTTDTTKERRI
SDAATPGASWHNANANAPPAGTPKTRDPYSYSQTADQHTPSYYNGGSYTSPRGPSYNRPRSPLSHSHQNPPAGSL
SPPGQQPLLASPSVRHGTTANMNPTTNGASAIPPFKSDLAAPSPPKPAPSSTTSRANPMSFDSILSSSEPAPKPK
EPSPIIAREPEIKEEREPRRDRESKRDSREPKQTKRSLEPELDHDTEVEKDVETEPEPLPSREKEKEPAPKKRGA
RKSTKGRASDIRDAATPKNGRRLSVKKESPTPRLPAKRQANGQPKPKTWSAEMEKKIQNAESDIENRAANLDADE
FDEQQYKERAQKRRRVMSELDVEYGLSRRDALANTISKKLVLHAELGKRRYDDVFYDEALHEVREQEVYAEKERK
KDMQRKRRREKSMAVTMEQKEAALARAEAAEDETERQKHLRDAERASKKAQQTKLILQKGIKGPARNLEINLEGG
TMSSFQASDVESGEAGTPSGKRKGKGRSGPRLKKSKEQKQAEKDSAEAAQAALDAGEELPTKEENRVRIKIKKTK
KDVAVDSEKDKDEAEKTEEEVVEKKTKKSKDKDKEKVDDIPDNEKRFMSKGYNQIYDQIWRDMARKDVNKTFKLA
VDSYATKASNLKKTAILASKEAKRWQLRTNKGTKDLQARAKRVMRDMMGFWKRNEREERDLRKAAEKQEIENARK
EEADREAARQKRKLNFLISQTELYSHFIGKKIKTDEVERSTDNPEIAKDAHQTDQKMLDIDEPTGPVIGKVTNFE
NLDFEEGSDEALRAAAMANAQNAIAEAQKKARDFNNQGLDMDDEGEMNFQNPTGLGDVEIEQPKLINAQLKEYQL
KGLNWLVNLYEQGINGILADEMGLGKTVQSISVMAYLAEKHDIWGPFLVVAPASTLHNWQQEIAKFVPEFKILPY
WGGASDRKVLRKFWDRKHTTYRKDAPFHVCVTSYQLVVSDVAYFQKMRWQYMILDEAQAIKSSQSSRWKALLNFH
CRNRLLLTGTPIQNNMQELWALLHFIMPSLFDSHDEFSEWFSKDIESHAQSNTKLNEDQLKRLHMILKPFMLRRV
KKHVQKELGDKIELDIFCDLTYRQRAYYSNLRNQINIMDLVEKATMGDDQDSGTLMNLVMQFRKVCNHPDLFERA
EVNSPFACAYFAETASFVREGNDVAVGYSSRNLIEYELPRLVWRDGGRVHKAGPDSQVAGWKNRTLNHLMNIWSP
DNIRDSSDGSKAFSWLRFADTSPNEAYQATHQSLIARAAKELQKRDRLGYMNVAYSDTEDANFTPAHALFQIRPR

QNRKPLADITNEGILSRLMNVAQGDYDESGLGRLEPAGRPRASAPPIQVSCRSWASEFERSEVLFNAPIRKILYG
PTVFEEKALVEKKLPMELWPTRQMLPKPDHEKKGFTNISIPSMQRFVTDSGKLAKLDDLLFKLKSEGHRVLLYFQ
MTRMIDMMEEYLTYRNYKYCRLDGSTKLEDRRDTVHDFQTRPEIFIFLLSTRAGGLGINLTTADTVIFYDSDWNP
TIDSQAMDRAHRLGQTKQVTVYRLITRGTIEERIRKRAMQKEEVQRVVIQGGGASVDFSGRRAPENRNRDIAMWL
ADDEQAEMIERREKELLESGELEKQQKKKGGKRRKAENSASLDEMYHEGEGNFDDGSKGVSGTATPATAATPADS
DSKGKKGRKGTKRAKTAKQRLAIADGMME
>Aspergillus_nigerGL06180_euk gi145245695refXP_001395110.1 helicase wr1
[Aspergillus niger CBS] -RKRRR
MQNGSPNGISHQDERVTPNLEGLPTDLPASEPPIESLSRDSTSADLKEEDGTTTAVDQLEGPPSKKRKLAGPSSS
RRSTSRPASPPWKKAGVDGPTSFIQDGRRKSSRVNAIPLQLQSPSEKRSTRGAQNKVIGRNVSGATKGVASSPLS
MSPPQPEPNGRLAGGSAAVNGSPRNATTRGSASRRHQISQSPAPKQSHTRTRSYSSGTARRSSNIGTASSTRSHR
SSMNNISTTMAETGDGDFDNAEDDEEYGQQAPRLRIKVKRPPIGIQHPGHVLAPRKYGSFKEWLESDDGRIRDPS
VLTPADALEEALKRRRIAVAAEPGGLLSPDVCSAYLPEQQEEPPQQYSHQDHLVAHALYFKKLLDQEHRRHRNTA
KLFAQWCADAWRKRNKDPEDILREQQEEMRGKRKQLAKDLQKMFDLARAEVDRMRLARWEEERKAEDQQALDRAI
KQSTMLFEKRRMEILGEGGSDAPDTSDAEQEDSDELLGGSEDEDNMSSTDSESEDENNVDDDEGLTAEELRLKYA
NLPNTNHDSDHESVVSDATAFSDESDTSEVDRSVDRATESLDPSAEVQPELEDVDPVLLDDSEDESTDMDDDMGD
TDEDESDEPDSDAESDGPGLLGFFSSKDIATNYGGSQSDVDGGDNAIIDDLDTKLASAGEDGEEEEEPEDPDEVS
LVPNGPALEMSILNDEEEAVSAPEPDPAPTSPSAPDSAVHLTADAEPKAAVESASELTPAPTPAADKTPDVEMTD
ASYNGDAVLPDASDAVAQTNPHEEHEHTRHEAYQSGEPSSEASPGTLVTKPSEPESISSYETPEKHPQPSESPAP
GLKTPVPHLLRGTLREYQHFGLDWLAGLYTNHINGILADEMGLGKTIQTIALLAHLAVEHEVWGPHLVVVPTSVI
LNWEMEFKKWCPGFKIMTYYGNQEERRQKRKGWMDDTSWNVLITSYQLVLQDQQVLKRRSWHYMILDEAHNIKNF
RSQRWQALLTFRTRARLLLTGTPLQNNLTELWSLLFFLMPSDGDEEGIEGFADLRNFSEWFRRPVEQILEHGRET
MDDEAKRVVTKLHTVLRPYILRRLKADVEKQMPAKYEHVVYCRLSKRQRFLYDGFMSRAQTKETLASGNYLSIIN
CLMQLRKVCNHPDLFETRPISTSFAMSRSVVTEYEIKDLLVRRRLLYEHPLTKLDLDFLNLVPISREDISRRLAD
DSTRLMAYGPFNTLRERQYHRTNWQMSFDGTTVQSTLDALENESRKRRMAELERCLYFESKRHGRRPVYGSSLIE
FLTADSKQRPTANGPLRKRSLADWLSSQSSVLASMILSIEERSQAMDGYVQRFACVTPAAVATGITEAALTPIET
RYLTKKERFPPYDPFHEAQMRLSIAFPDKRLLQYDCGKLQRLDKLLRDLKAGGHRALIFTQMTKMLDILEQFLNI
HGHRYLRLDGTTKVEQRQILTDRFNNDNRILAFILSSRSGGLGINLTGADTVIFYDLDWNPAMDKQCQDRCHRIG
QTRDVHIYRFVSEYTIESNILRKANQKRMLDDVVIQEGEFTTDYFTKLDVRDMIDTEDVLEGHDEASAAMDRVLE
NRVAASSRVFEQAEDKEDIDAAKNAQKELEHADDGDFDDRTPGQTQAGTPLATGPTTGPDESTTANTTPGPQLLT
SPQVHAVDEPVDVEPQPGHIDNYLLRFMEWNMKDEPLVLPADKSKKKSKKGKEHRLRKRRR
>Aspergillus_nigerGL09156_euk gi145253731refXP_001398378.1 DNA helicase ino80
[Aspergillus niger CBS] -RKRRR
MTGAPPYNPQSPTQQSRYPVYSPPAKSRPYYANNEQYQQHPPQTPPAFSSRSPHFSHAPSPLPGTLPPLNGAAPP
SSHPSEPPSQYQAHSSAGNPQFALPRPYPGSVLSGNGASPYGHSTPSHAHPAGRPDSHPQTSPKKESESQFPMST
HGVMGYPSSVVREPRASSPPKDVKPTRAADPMSFASILSGPTEERAPPPRQSSAEATPTPLAPAATAQTSLSPPP
VASAATSQKSKDRAPALTASLPRLEKKPTTEKRRRNPPETEQKTADSRTSNVANGVSEPSKTLAQPRGAGSRPVM
SERETEALNKALADMAEADKSDVEAPGYDRFREEYRAKGKKRAFATEQAEILRRKRRRNDFLVKLSKSLEKQATA
GMDRFRYANEASVVAEVQAKEIQDEKERKKDMQRKRRRENTVRMEMQKKKEAEAKAHEAQDSAEKAKFLREAERA
QRKIKTTKRALEGITAPEEISEVTPLAPNLEGGTTSSFHIGRGSPSRRKSGRGGPVTRPKKSKEQKQAEKDAAEA
AYAAMENDEPLPIAPKEDPRKESLKKEVKGGRSKEPTPTPLSAYETKGYNQIYEQIWRDIARKDIPKVYRIKALS
LSTRQENLRKTAQLASKQSRKWQERTNKSMKDTQARAKRTMREMMSFWKRNEREERDLRRLAEKQEIESAKKAEA
EREANRQRRKLNFLISQTELYSHFIGRKIKGAEGDAAGDTAVEATGETVQPGKGQDHTIDMPSSVADAGTKVTNF
EDLDFDAEDETALRQAAMANAQNAVQEAQDRARAFNSGQNQMDALDEGELNFQNPTSLGDIEISQPNMLTAKLKE

YQLKGLNWLVNLYEQGINGILADEMGLGKTIQSISVMAYLAEVHNIWGPFLVIAPASTLHNWQQEITKFVPDIKV
LPYWGSAKDRKILRKFWDRKHITYTKESEFHVLVTSYQLVVLDAQYFQKVKWQYMILDEAQAIKSSQSSRWKNLL
GFHCRNRLLLTGTPIQNNMQELWALLHFIMPTLFDSHDEFSEWFSKDIESHAQSNTKLNEDQLRRLHMILKPFML
RRVKKHVQQELGDKVEKDIFCDLTYRQRAYYTNLRNRVSIMDLIEKAAVGDEADSTTLMNLVMQFRKVCNHPDLF
ERAETKSPFSTAYFAETASFVREGNNVDVRYSTRNLIEYPMPRLLCGAGGRVDVAGAENPHAGFRGRYLNHLMNI
FTPENMKQSIQDDGAFSFLRFVDTSLGEAYEQSHLGIFERAVRRRGQVNRLSRLNVAYDDDKELAGSALPHTLFN
IVDRNDKHAVNEVAAEGIMRDLMTVSQSTYEREGLNIIEPCVSPAASAPPISVVSSSHIPSIETRDTLFNVSVRH
ALYSTPSRQVDEQIIEKKVDPTPYSLAPMLPKPISAKGRYTHIEVPSMRRFVTDSGKLAKLDELLRELKAGGHRV
LLYFQMTRMIDLMEEYLTYRNYKYCRLDGSTKLEDRRDTVADFQQRPEIFVFLLSTRAGGLGINLTAADTVIFYD
SDWNPTIDSQAMDRAHRLGQTRQVTVYRLITRGTIEERIRKRALQKEEVQRVVITGGAAGGVDFNTRNRESRTKD
IAMWLADDEQAELIEQKEKEALDRGEVFGAGKGGKKAAQKRKKDITLDDMYHEGEGNFDDASAKPSGAATPVSTA
ENVGTPSSTPAPKRGRGRGSGKGTSKRAKTTKERLRLIDGDGGLGPS

Dataset S2. NES-bearing proteins in the predicted protein pools of the 27 strains.

NES motif: -SLPHAILRIDLA

>Eremothecium_cymbalariaeGL00481 gi363748682refXP_003644559.1 hypothetical protein Ecym_1520

MDSGEVAALVIDNGSGMCKAGFAGDDAPRAVFPSIVGRPRHQGIMVGMGQKDCYVGDEAQSKRGILTLRYPIEHG
IVTNWDDMEKIWHHTFYNELRVAPEEHPVLLTEAPMNPKSNREKMTQIMFETFNVPAFYVSIQAVLSLYSSGRTT
GIVLDSGDGVTHVVPIYAGFSLPHAILRIDLAGRDMTDYLMKILSERGYSFSTTAEREIVRDIKEKLCYVALDYD
QEMQTAAQSSAIEKSYELPDGQVITIGNERFRAPEALFHPSVLGLEAAGIDQTTYNSIMKCDVDVRKELYGNIVM
SGGTTMFPGIAERMQKEITALAPSSMKVKIIAPPERKYSVWIGGSILASLTTFQQMWISKQEYDESGPSIVHHKC
F

>Saccharomyces_cerevisiaeGL01721 gi|14318479|ref|NP_116614.1| Act1p

MDSEVAALVIDNGSGMCKAGFAGDDAPRAVFPSIVGRPRHQGIMVGMGQKDSYVGDEAQSKRGILTLRYP
IEHGIVTNWDDMEKIWHHTFYNELRVAPEEHPVLLTEAPMNPKSNREKMTQIMFETFNVPAFYVSIQAVL
SLYSSGRTTGIVLDSGDGVTHVVPIYAGFSLPHAILRIDLAGRDLTDYLMKILSERGYSFSTTAEREIVR
DIKEKLCYVALDFEQEMQTAAQSSSIEKSYELPDGQVITIGNERFRAPEALFHPSVLGLESAGIDQTTYN
SIMKCDVDVRKELYGNIVMSGGTTMFPGIAERMQKEITALAPSSMKVKIIAPPERKYSVWIGGSILASLT
TFQQMWISKQEYDESGPSIVHHKCF

NES motif: -DIKEKLCYVALD

>Saccharomyces_cerevisiaeGL01721 gi|14318479|ref|NP_116614.1| Act1p

MDSEVAALVIDNGSGMCKAGFAGDDAPRAVFPSIVGRPRHQGIMVGMGQKDSYVGDEAQSKRGILTLRYP
IEHGIVTNWDDMEKIWHHTFYNELRVAPEEHPVLLTEAPMNPKSNREKMTQIMFETFNVPAFYVSIQAVL
SLYSSGRTTGIVLDSGDGVTHVVPIYAGFSLPHAILRIDLAGRDLTDYLMKILSERGYSFSTTAEREIVR
DIKEKLCYVALDFEQEMQTAAQSSSIEKSYELPDGQVITIGNERFRAPEALFHPSVLGLESAGIDQTTYN
SIMKCDVDVRKELYGNIVMSGGTTMFPGIAERMQKEITALAPSSMKVKIIAPPERKYSVWIGGSILASLT
TFQQMWISKQEYDESGPSIVHHKCF

>Aspergillus_nigerGL07818 gi317035293refXP_003188912.1 actin

MEEEVAALVIDNGSGMCKAGFAGDDAPRAVFPSIVGRPRHHGIMIGMGQKDSYVGDEAQSKRGILTLRYPIEHGV
VTNWDDMEKIWHHTFYNELRVAPEEHPVLLTEAPINPKSNREKMTQIVFETFNAPAFYVSIQAVLSLYASGRTTG
IVLDSGDGVTHVVPIYEGFALPHAISRVDMAGRDLTDYLMKILAERGYTFSTTAEREIVRDIKEKLCYVALDFEQ
EIQTASQSSSLEKSYELPDGQVITIGNERFRAPEALFAPSVLGLESGGIHETTFNSIMKCDVDVRKDLYGNIVMS
GGTTMYPGISDRMQKEITALAPSSMKVKIIAPPERKYSVWIGGSILASLSTFQQMWISKQEYDESGPSIVHRKCF

>Aspergillus_nigerGL07819 gi145250163refXP_001396595.1 actin

MEEEVAALVIDNGSGMCKAGFAGDDAPRAVFPSIVGRPRHHGIMIGMGQKDSYVGDEAQSKRGILTLRYPIEHGV
VTNWDDMEKIWHHTFYNELRVAPEEHPVLLTEAPINPKSNREKMTQIVFETFNAPAFYVSIQAVLSLYASGRTTG
IVLDSGDGVTHVVPIYEGFALPHAISRVDMAGRDLTDYLMKILAERGYTFSTTAEREIVRDIKEKLCYVALDFEQ
EIQTASQSSSLEKSYELPDGQVITIGNERFRAPEALFAPSVLGLESGGIHETTFNSIMKCDVDVRKDLYGNIVMS
GGTTMYPGISDRMQKEITALAPSSMKVKIIAPPERKYSVWIGGSILASLSTFQQMWISKQEYDESGPSIVHRKCF

>Eremothecium_cymbalariaeGL00481 gi363748682refXP_003644559.1 hypothetical protein Ecym_1520

MDSGEVAALVIDNGSGMCKAGFAGDDAPRAVFPSIVGRPRHQGIMVGMGQKDCYVGDEAQSKRGILTLRYPIEHG
IVTNWDDMEKIWHHTFYNELRVAPEEHPVLLTEAPMNPKSNREKMTQIMFETFNVPAFYVSIQAVLSLYSSGRTT
GIVLDSGDGVTHVVPIYAGFSLPHAILRIDLAGRDMTDYLMKILSERGYSFSTTAEREIVRDIKEKLCYVALDYD

QEMQTAAQSSAIEKSYELPDGQVITIGNERFRAPEALFHPSVLGLEAAGIDQTTYNSIMKCDVDVRKELYGNIVM
SGGTTMFPGIAERMQKEITALAPSSMKVKIIAPPERKYSVWIGGSILASLTTFQQMWISKQEYDESGPSIVHHKC
F
>Gibberella_zeaeGL07455 gi|46125915|ref|XP_387511.1| ACTG_CEPAC Actin
MTYLCFTEEVAALVIDNGSGMCKAGFAGDDAPRAVFPSIVGRPRHHGIMIGMGQKDSYVGDEAQSKRGILTLRYP
IEHGVVTNWDDMEKIWHHTFYNELRVAPEEHPVLLTEAPINPKSNREKMTQIVFETFNAPAFYVSIQAVLSLYAS
GRTTGIVLDSGDGVTHVVPIYEGFALPHAIARVDMAGRDLTDYLMKILAERGYTFSTTAEREIVRDIKEKLCYVA
LDFEQEIQTAAQSSSLEKSYELPDGQVITIGNERFRAPEALFQPSVLGLESGGIHVTTFNSIMKCDVDVRKDLYG
NIVMSGGTTMYPGLSDRMQKEITALAPSSMKVKIIAPPERKYSVWIGGSILASLSTFQQMWISKQEYDESGPSIV
HRKCF
>Penicillium_chrysogenum_WisconsinGL07192 gi255945763refXP_002563649.1
gamma-actin act
MEEEVAALVIDNGSGMCKAGFAGDDAPRAVFPSIVGRPRHHGIMIGMGQKDSYVGDEAQSKRGILTLRYPIEHGV
VTNWDDMEKIWHHTFYNELRVAPEEHPILLTEAPINPKFNREKMTQIVFETFNAPAFYVSIQAVLSLYASGRTTG
IVLDSGDGVTHVVPIYEGFSLPHAISRVDMAGRDLTDYLMKILAERGYTFSTTAEREIVRDIKEKLCYVALDFEQ
EIQTASQSSSLEKSYELPDGQVITIGNERFRAPEALFQPNVLGLESGGIHVTTFNSIMKCDVDVRKDLYGNIVMS
GGTTMYPGISDRMQKEITALAPSSMKVKIIAPPERKYSVWIGGSILASLSTFQQMWISKQEYDESGPSIVHRKCF

NES motif: -IEAALSDALAALQI
>Saccharomyces_cerevisiaeGL00680 gi|6319972|ref|NP_010052.1| Ssb1p
MAEGVFQGAIGIDLGTTYSCVATYESSVEIIANEQGNRVTPSFVAFTPEERLIGDAAKNQAALNPRNTVFDAKRL
IGRRFDDESVQKDMKTWPFKVIDVDGNPVIEVQYLEETKTFSPQEISAMVLTKMKEIAEAKIGKKVEKAVITVPA
YFNDAQRQATKDAGAISGLNVLRIINEPTAAAIAYGLGAGKSEKERHVLIFDLGGGTFDVSLLHIAGGVYTVKST
SGNTHLGGQDFDTNLLEHFKAEFKKKTGLDISDDARALRRLRTAAERAKRTLSSVTQTTVEVDSLFDGEDFESSL
TRARFEDLNAALFKSTLEPVEQVLKDAKISKSQIDEVVLVGGSTRIPKVQKLLSDFFDGKQLEKSINPDEAVAYG
AAVQGAILTGQSTSDETKDLLLLDVAPLSLGVGMQGDMFGIVVPRNTTVPTIKRRTFTTCADNQTTVQFPVYQGE
RVNCKENTLLGEFDLKNIPMMPAGEPVLEAIFEVDANGILKVTAVEKSTGKSSNITISNAVGRLSSEEIEKMVNQ
AEEFKAADEAFAKKHEARQRLESYVASIEQTVTDPVLSSKLKRGSKSKIEAALSDALAALQIEDPSADELRKAEV
GLKRVVTKAMSSR
>Saccharomyces_cerevisiaeGL04592 gi|6324120|ref|NP_014190.1| Ssb2p
MAEGVFQGAIGIDLGTTYSCVATYESSVEIIANEQGNRVTPSFVAFTPQERLIGDAAKNQAALNPRNTVFDAKRL
IGRRFDDESVQKDMKTWPFKVIDVDGNPVIEVQYLEETKTFSPQEISAMVLTKMKEIAEAKIGKKVEKAVITVPA
YFNDAQRQATKDAGAISGLNVLRIINEPTAAAIAYGLGAGKSEKERHVLIFDLGGGTFDVSLLHIAGGVYTVKST
SGNTHLGGQDFDTNLLEHFKAEFKKKTGLDISDDARALRRLRTAAERAKRTLSSVTQTTVEVDSLFDGEDFESSL
TRARFEDLNAALFKSTLEPVEQVLKDAKISKSQIDEVVLVGGSTRIPKVQKLLSDFFDGKQLEKSINPDEAVAYG
AAVQGAILTGQSTSDETKDLLLLDVAPLSLGVGMQGDIFGIVVPRNTTVPTIKRRTFTTVSDNQTTVQFPVYQGE
RVNCKENTLLGEFDLKNIPMMPAGEPVLEAIFEVDANGILKVTAVEKSTGKSSNITISNAVGRLSSEEIEKMVNQ
AEEFKAADEAFAKKHEARQRLESYVASIEQTVTDPVLSSKLKRGSKSKIEAALSDALAALQIEDPSADELRKAEV
GLKRVVTKAMSSR

NES motif: -FKPDMNPALREVLEALEDEAYVVNDD
>Saccharomyces_cerevisiaeGL03264 gi|6322706|ref|NP_012779.1| Ltv1p
MSKKFSSKNSQRYVVVHRPHDDPSFYDTDASAHVLVPVSNPNKTSPEADLRKKDVSSTKPKGRRAHVGEAALYGI
NFDDSEYDYTQHLKPIGLDPENSIFIASKGNEQKVEKKNIEDLFIEPKYRRDEIEKDDALPVFQRGMAKPEYLLH

QQDTTDEIRGFKPDMNPALREVLEALEDEAYVVNDDVVVEDISKKTQLQGDNYGEEEKEDDIFAQLLGSGEAKDE
DEFEDEFDEWDIDNVENFEDENYVKEMAQFDNIENLEDLENIDYQADVRRFQKDNSILEKHNSDDEFSNAGLDSV
NPSEEEDVLGELPSIQDKSKTGKKKRKSRQKKGAMSDVSGFSMSSSAIARTETMTVLDDQYDQIINGYENYEEEL
EEDEEQNYQPFDMSAERSDFESMLDDFLDNYELESGGRKLAKKDKEIERLKEAADEVSKGKLSQRRNRERQEKKK
LEKVTNTLSSLKF

NES motif: -QLLQEKLEKLTKLK
>Saccharomyces_cerevisiaeGL01983 gi|6321352|ref|NP_011429.1| Mad1p
MDVRAALQCFFSALSGRFTGKKLGLEIYSIQYKMSNSGGSSPFLESPGGSPDVGSTNGQSNRQIQALQFKLNTLQ
NEYEIEKLQLQKQTNILEKKYKATIDELEKALNDTKYLYESNDKLEQELKSLKERSANSMNDKDKCIEELRTTLQ
NKDLEMETLRQQYDSKLSKVTNQCDHFKLEAESSHSLLMKYEKEIKRQSVDIKDLQHQVMEKDDELSSVKASKMI
NSHPNYSTEEFNELTEMNKMIQDQVQYTKELELANMQQANELKKLKQSQDTSTFWKLENEKLQNKLSQLHVLESQ
YENLQLENIDLKSKLTKWEIYNDSDDDDDNNVNNNDNNNNNKNDNNNDNNNDTSNNNNINNNNRTKNNIRNNPEE
IIRDWKLTKKECLILTDMNDKLRLDNNNLKLLNDEMALERNQILDLNKNYENNIVNLKRLNHELEQQKSLSFEEC
RLLREQLDGLYSAQNNALLEVENSETHASNKNVNEDMNNLIDTYKNKTEDLTNELKKLNDQLLSNSNDVETQRKK
RKLTSDQIGLNYSQRLNELQLENVSVSRELSKAQTTIQLLQEKLEKLTKLKEKKIRILQLRDGPFIKDQFIKKNK
LLLLEKENADLLNELKKNNPAVETVPISVYDSLNFELKQFEQEVFKSNKRFSRLKQVFNNKSLEFIDVVNSLLGF
KLEFQQDSRVKIFSCFKPEKYLIADLNENTLKSNLDADIEGWDDLMNLWVEDRGQLPCFLATITLRLWEQRQAK

NES motif: -INIDELLDEL
>Saccharomyces_cerevisiaeGL02572 gi|6321964|ref|NP_012040.1| Nmd3p
MEFTPIDPHQHQNAATLLCCNCGTPIDGSTGLVMCYDCIKLTVDITQGIPREANISFCRNCERFLQPPGQ
WIRAELESRELLAICLRRLKGLTKVRLVDASFIWTEPHSRRIRIKLTVQGEAMTNTIIQQTFEVEYIVIA
MQCPDCARSYTTNTWRATVQIRQKVPHKRTFLFLEQLILKHNAHVDTISISEAKDGLDFFYAQKNHAVKM
IDFLNAVVPIKHKKSEELISQDTHTGASTYKFSYSVEIVPICKDDLVVLPKKLAKSMGNISQFVLCSKIS
NTVQFMDPTTLQTADLSPSVYWRAPFNALADVTQLVEFIVLDVDSTGISRGNRVLADITVARTSDLGVND
QVYYVRSHLGGICHAGDSVMGYFIANSNYNSDLFDGLNIDYVPDVVLVKKLYQRKSKKSRHWKLKRMAKE
HKDIDASLDYNSRAQKQEMERAEKDYELFLQELEEDAELRQSVNLYKNREANVPPEEHEMDEDEDEDAPQ
INIDELLDELDEMTLEDGVENTPVESQQ
>Eremothecium_cymbalariaeGL02620 gi363752964refXP_003646698.1 hypothetical protein Ecym_5097
MNYTPLDENHMQHKAATVLCCNCGVPMDGSTGLVMCYDCIKLTVDITEGIPREANVSFCRNCERFLQPPGQWIRA
ELESRELLALCLRRLKGLNKVRLVDASFIWTEPHSRRIRIKLTVQGEAMANTIIQQTFEVEYIVVAMQCPDCARS
YTANTWRANIQIRQKVPHKRTFLYLEQLILKHNAHVDTVSISEAKDGLDFYYSQKNHAVKMLDFLNSVVPVKTKK
SEELISQDTHTGASTYKFSYSVEIVPICRDDLVVLPKKLAKSMGNISQFVICSKVTNALQFLDPQTLQTAELTAS
VYWRNPFMSLADVSQLIEFIVLEVEPTGYVNGKRVLADITVARASDMGVNDQTYYVRSHLGAICHPGDSVMGYFI
ANSNYNSDLFDSLNFDRIPDIVLVKKQYIRKTRKNRHWKLKRMAKEHKDIDASNDYSRQQKVDMERAERDYELFL
QELEEDEEMRQTINLYKNQKPVQEEEMADSEDEDAPQINIDELLDELDEMTLDDLTTAE
